# Specific Targeting of Lymphoma Cells Using Semisynthetic Anti-Idiotype Shark Antibodies

**DOI:** 10.3389/fimmu.2020.560244

**Published:** 2020-11-26

**Authors:** Arturo Macarrón Palacios, Julius Grzeschik, Lukas Deweid, Simon Krah, Stefan Zielonka, Thies Rösner, Matthias Peipp, Thomas Valerius, Harald Kolmar

**Affiliations:** ^1^ Institute for Organic Chemistry and Biochemistry, Technische Universität Darmstadt, Darmstadt, Germany; ^2^ Division of Stem Cell Transplantation and Immunotherapy, Department of Medicine II, UKSH, CAU Kiel, Kiel, Germany

**Keywords:** B-cell receptor, idiotype, lymphoma, yeast display, vNAR, antibody-drug conjugate

## Abstract

The B-cell receptor (BCR) is a key player of the adaptive immune system. It is a unique part of immunoglobulin (Ig) molecules expressed on the surface of B cells. In case of many B-cell lymphomas, the tumor cells express a tumor-specific and functionally active BCR, also known as idiotype. Utilizing the idiotype as target for lymphoma therapy has emerged to be demanding since the idiotype differs from patient to patient. Previous studies have shown that shark-derived antibody domains (vNARs) isolated from a semi-synthetic CDR3-randomized library allow for the rapid generation of anti-idiotype binders. In this study, we evaluated the potential of generating patient-specific binders against the idiotype of lymphomas. To this end, the BCRs of three different lymphoma cell lines SUP-B8, Daudi, and IM-9 were identified, the variable domains were reformatted and the resulting monoclonal antibodies produced. The SUP-B8 BCR served as antigen in fluorescence-activated cell sorting (FACS)-based screening of the yeast-displayed vNAR libraries which resulted after three rounds of screening in the enrichment of antigen-binding vNARs. Five vNARs were expressed as Fc fusion proteins and consequently analyzed for their binding to soluble antigen using biolayer interferometry (BLI) revealing binding constants in the lower single-digit nanomolar range. These variants showed specific binding to the parental SUP-B8 cell line confirming a similar folding of the recombinantly expressed proteins compared with the native cell surface-presented BCR. First initial experiments to utilize the generated vNAR-Fc variants for BCR-clustering to induce apoptosis or ADCC/ADCP did not result in a significant decrease of cell viability. Here, we report an alternative approach for a personalized B-cell lymphoma therapy based on the construction of vNAR-Fc antibody-drug conjugates to enable specific killing of malignant B cells, which may widen the therapeutic window for B-cell lymphoma therapy.

## Introduction

In recent years, monoclonal antibodies have emerged as therapeutics with exceptional relevance for inflammatory and infectious diseases and the treatment of cancer (1). The number of antibodies in late-stage clinical trials has substantially increased to over 50 in the year 2017. Furthermore, in this year the number of novel antibody therapeutics that were approved in either United States or the European Union reached double-digit numbers for the first time (2). Being approved in 1997, the monoclonal antibody (mAb) Rituximab was the first FDA approved mAB specifically for cancer treatment and was for a long time the most sold biologic drug in clinical oncology. Rituximab is active in a diversity of human lymphomas and chronic lymphocytic leukemia (3). Rituximab is a chimeric mAB targeting the CD20 antigen present on both healthy B cells as well as on most B-cell lymphomas.

Today 5% of all newly diagnosed malignancies are lymphoma-related (4). More than 90% of adult lymphomas are mature B-cell non-Hodgkin lymphomas (B-NHL), with a large variety of histological subtypes (5). Besides Rituximab, a plethora of different small molecules and mAbs have been developed that either target different CD20 epitopes (for instance ofatumumab and obinutuzumab) or other B-cell specific antigens as CD19, CD22, and CD30 (6–10). For B-cell targeting with increased efficacy, the effect of antibody-drug conjugates (ADCs) has been analyzed in clinical trials (11). The mentioned different antibody therapies share the characteristic of targeting B cells in a non-specific manner. CD20 and other potential surface markers are present on B cells regardless of the targeted cell being malignant or not. Thus, all of the approved therapeutics can lead to severe side effects (12). As a consequence, the specific targeting of tumor cells might emerge as beneficial for lymphoma therapies (13, 14).

Most B-cell lymphomas express a cell surface immunoglobulin molecule referred to as B-cell receptor (BCR). The BCR consists of a membrane-anchored immunoglobulin (Ig) associated with transmembrane proteins. The extracellular region of the BCR is an antibody normally serving as recognition site for foreign antigens (15). It has a crucial role in signaling after first encounter of a B cell with its cognate antigen. Here, the BCR mediates antigen internalization and the subsequent presentation of peptides to T-helper cells. This results in triggering of cell proliferation, differentiation and the generation of memory B cells and antibody-secreting B cells (16).

In general, B-cell lymphomas develop from clonal populations of precursor B cells in different stages of B-cell differentiation. Consequently, the BCR is different for each lymphoma patient since variable regions of BCRs are generated by random rearrangement of germ line immunoglobulin genes (17). During malignant transformation of a B cell, each cell of the formed clonal population expresses the same unique BCR, the so-called idiotype (Id).

Specific binding can not only be utilized for specific cellular targeting but also for induction of anergy and clonal deletion of lymphoma cells. This occurs when B-cells perceive a BCR stimulus in the absence of T-cell help (18), Toll-like receptor binding or cytokine signals from accessory cells (19). Consequently, the BCR might act as a tumor-specific and functionally active cell surface marker able to trigger apoptosis.

Previous studies have shown the general applicability of mAbs directed against the patient-specific Ids in lymphoma studies (20). Although these studies have confirmed the safe administration and positive immunological effects *in-vivo*, the strategy of Id-targeting has not emerged as a widespread therapy for lymphomas until now. The most important limiting factor was the requirement to generate tailor-made mAbs for every single patient. Since the application of classical hybridoma technology (21) for producing individualized mAbs was not feasible in a cost-effective process, this strategy was not further investigated extensively in the following years. The group of Ron Levy, one of the pioneers of anti-Id mAbs for lymphoma treatment (20, 22), brought the issue of anti Id-mAbs back on the agenda: They published a new concept for patient-specific lymphoma targeting based on anti-Id peptides in 2016 (23).

The respective peptides specifically recognizing the BCR were identified by screening a phage-display library (24), chemically synthesized, and then coupled chemically to the amino terminus of a premade IgG Fc protein. They clearly demonstrated the specific recognition of tumor cells *in vitro*, confirmed the triggering of tumor cell apoptosis and phagocytosis by macrophages, and effectively cleared human lymphoma cells in a murine xenograft model. However, utilizing peptides for specific BCR-binding might have some drawbacks, for example a short half-life *in-vivo* and their significant unspecific binding after administration that might occur. To evaluate alternatives to this strategy, we decided to use shark-derived antibody fragments for the generation of anti-Id binders.

Compared to conventional antibodies from mammals, cartilaginous fish like sharks have a unique form of heavy chain-only antibodies, termed immunoglobulin new antigen receptor (IgNAR) (25). Molecules of this type are devoid of any light chain but possess heavy chains comprised of five constant domains followed by the variable domain (vNAR) that is exclusively responsible for antigen binding. Since vNARs lack the hydrophobic V_H_–V_L_ interface of a conventional antibody, they are reported to have an increased water solubility (26, 27). Furthermore, vNARs only possess two (CDR1 and CDR3) CDR loops leading to the fact that vNARs, with a size of 11 to 12 kDa, are the smallest antigen-binding antibody-like domain in the animal kingdom (28). Of note, the elongated CDR3 loop primarily mediating antigen-binding is at least partly able to compensate for the lack of the CDR2 loop. Additionally, it is reported that vNARs, based on their elongated CDR3 loop, are able to interact with hidden, cleft-like epitopes not able to be recognized by conventional antibodies. Besides the isolation of vNARs directed against therapeutically relevant target proteins as TNFα (29), the malaria-related AMA protein (30) or EpCAM (31), it has been shown that vNARs can be engineered towards pH-sensitive antigen-binding (32) for application in biotechnological applications. Recently, it was shown that screening of semi-synthetic and CDR3-randomized vNAR libraries in combination with yeast surface display allows for the fast and convenient identification of anti-idiotypic vNAR domains directed against the paratope of mAbs (33). The resulting anti-idiotypic vNAR variants were not cross-reactive towards unrelated antibodies and retained their excellent target recognition in the presence of human and murine serum.

**Figure 1:**
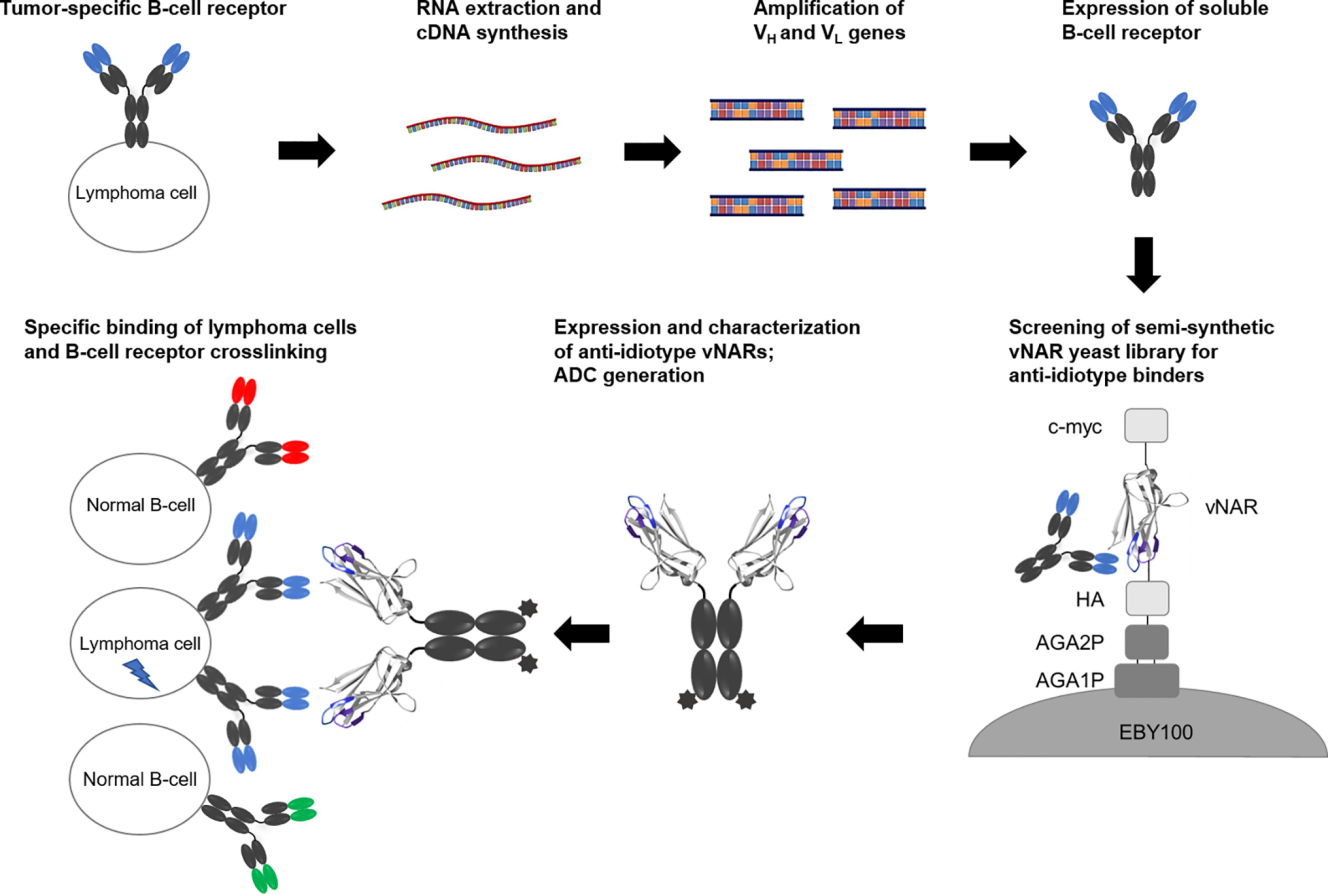
Concept overview. RNA of B cells presenting a tumor-specific B-cell receptor is extracted followed by cDNA synthesis. After amplification of genes coding for variable domains, soluble BCR molecules are expressed in mammalian cells. A semi-synthetic shark-derived vNAR yeast library is screened to identify anti-idiotype binders. After expression as vNAR-Fc molecules, these candidates are analyzed for their specific cellular binding and their potential to induce cell death.

Based on these experimental experiences, we investigated in this study whether this strategy can also be applied to the isolation of anti-idiotype vNARs specifically recognizing the BCR of lymphoma cell lines and finally inducing cell death (**Figure 1**). Here we show that yeast library screening for vNARs directed against soluble BCR SUP-B8 lymphoma cells results in anti-idiotype vNARs showing specific receptor binding. Since we could not observe the induction of apoptosis *via* clustering of BCR molecules in first initial experiments, we developed an alternative approach by generating vNAR antibody-drug conjugates that induced specific killing in lymphoma cells at low nanomolar concentrations.

## Materials and Methods 

Experiments with human effector cells were approved by the Ethics Committees of the participating institutions in accordance with the Declaration of Helsinki.

### Cell Lines, RNA Isolation, and cDNA Synthesis

Three immunoglobulin expressing lymphoma cell lines Daudi, IM-9, and SUP-B8 (obtained from the group of Prof. Ron Levy) were used for idiotype cloning. Daudi and IM-9 cells were grown in RPMI-1640 medium with 10% FBS and 2 mM glutamine. SUP-B8 cells were treated the same way except using 15% FBS for cultivation. Extraction of total RNA from 1 × 10^7^ human lymphoma cells was carried out after cell homogenization with QIAshredder (Qiagen) using the commercially available kit RNeasy Minikit (Qiagen) according to the supplier's protocol. Subsequently, cDNA-synthesis was carried out using 50 µl RNA extract, 10 µl random hexamer primers (50 ng/µl), 10 µl 50 µM dNTPs, 30 µl H_2_O, 40 µl 25 mM MgCl_2_, 20 µl 10× RT-buffer, 20 µl 0.1 M DTT, 10 µl RNase OUT, and 10 µl SuperScript III reverse transcriptase (all components acquired from SuperScript III First-Strand Kit, Thermo Fisher Scientific). Incubation for 5 min at 25°C was followed by incubation for 60 min at 50°C and an inactivation step at 85°C for 5 min. Subsequently, 1 µl RNAaseH was added and the reaction was incubated for 20 min at 37°C. The synthesized cDNA was stored at −20°C.

### Expression of Soluble B-Cell Receptors

Variable antibody regions were amplified from cDNA in two different PCR reactions using Taq or Q5 Polymerase (NEB) in a reaction volume of 100 µl. For each cell line and chain, the reactions were prepared using 1 to 2 µl cDNA. Amplification of V_H_ and V_L_ genes was carried out using the primers listed in Tab. S1. A detailed overview about the amplification strategy can be found in a publication by Maleshko et al. (34). For all reactions the following conditions were set: 95°C for 30 s, 30 cycles of 20 s at 95°C, 30 s at 57°C, and 30 s at 68°C, followed by 68°C for 5 min. PCR products were purified *via* Wizard® SV Gel and PCR Clean-up System (Promega). Afterwards PCR products were sent for sequencing by the company Seqlab. For cloning of B-cell receptors of all used cell line V_H_ genes were amplified using primers introducing restriction sites for *Bam*HI and *Apa*I (Daudi_HC_BamHI_up/IM9_HC_BamHI_up/SUPB8_HC_BamHI_up and Daudi_HC_ApaI_lo/IM9_HC_ApaI_lo/SUPB8_HC_ApaI_lo). For light chain cloning of Daudi and IM-9 cell lines V_L_ genes and the C_κ_ domain were amplified separately utilizing the primers Daudi_LC_BamHI_up/IM9_LC_BamHI_up and Daudi_LC_SOE_LO/IM9_LC_SOE_LO for V_L_ genes on the one hand and Daudi_LC_SOE_UP/IM9_LC_SOE_UP with LC_kappa_NotI_lo for C_κ_ on the other hand. For the latter reaction a vector bearing the coding sequence for Matuzumab kappa light chain served as template. Afterwards fusion PCR was performed using the primer pair IM9_LC_up/ Daudi_LC_up and LC_kappa_NotI_lo. 20 ng of each domain was amplified using Taq polymerase for 15 cycles without addition of oligonucleotides with an elongation time of 50 s. Subsequently, 15 PCR cycles were performed after addition of oligonucleotides. Afterwards the products were purified *via* Wizard® SV Gel and PCR Clean-up System. Light chain of SUPB8 was constructed in a similar way: After separate amplification of V_L_ genes using the primers SUPB8_LC_BamHI_up and SUPB8_LC_SOE_lo and the C_λ_ domain using the oligonucleotides SUPB8_LC_SOE_up and SUPB8_Lambda_NotI_lo. For the latter reaction the vector pYD_lambda_constant with the coding sequence for lambda light chain served as template. Subsequent fusion PCR and purification was performed according to the generation of Daudi and IM9 light chains. Restriction of PCR products and vectors pTT5-hu425H/pTT5-hu425L (NRC Biotechnology Research Institute) with the respective restriction enzymes was followed by ligation and transformation into *Escherichia coli* strain DH5α. After incubation overnight plasmids were isolated using the PureYield™ Plasmid Miniprep System (Promega) followed by sequence verification. After that Expi293F™ cells were transiently transfected using polyethyleneimine (Polysciences) according to the instructions of the manufacturer (Thermo Fisher Scientific). After 5 days the respective culture supernatants were harvested, sterile-filtered (0.22 μm Filtropur S 0.2, Sarstedt) and purified using HiTrap Protein A HP columns (GE Healthcare) in combination with an ÄKTA purification system (GE Healthcare). This was followed by buffer exchange *via* dialysis against PBS using a membrane with a MWCO ~ 14 kDa. The purified proteins were stored at −80°C.

### Yeast Strains, Media, and Reagents

*Saccharomyces cerevisiae* strain EBY100 was utilized for yeast surface display (35). YPD medium was composed of 20 g/L dextrose, 20 g/L tryptone, and 10 g/L yeast extract. SD-CAA medium was prepared using 8.6 g/L NaH_2_PO_4_ × H_2_O, 5.4 g/L Na_2_HPO_4_, 1.7 g/L yeast nitrogen base without amino acids, 5 g/L ammonium sulphate, 5 g/L bacto casamino acids, and 20 g/L dextrose. SG-CAA medium was prepared the same way except for the substitution of glucose sugar with galactose. Phosphate-buffered saline (PBS) was prepared with 8.1 g/L NaCl, 1.13 g/L Na_2_HPO_4_, 0.75 g/L KCl, and 0.27 g/L KH_2_PO_4_.

### Yeast Library Construction

The generation of semi-synthetic vNAR yeast display libraries was extensively described by Grzeschik et al. (36). Briefly, the PCR-amplified vNAR products from three non-immunized bamboo sharks were used as template for a consecutive three-step PCR. At first a cysteine in CDR1 was mutated to tyrosine to prevent the non-specific generation of disulfide bridges. In the second PCR, randomization of either 12, 14, 16 or 18 amino acids in the CDR3 was performed using a codon-based trinucleotide mixture (EllaBiotech) that consisted of each amino acid-encoding trimer except the one for cysteine. Performing the third PCR allowed for attachment of homologous sequences for gap repair cloning in yeast with the linearized pCT vector (35). PCR products were purified using the Wizard® SV Gel and PCR Clean-up System (Promega) according to the manufacturer's protocol. The pCT plasmid used for gap repair cloning and surface presentation of vNAR variants was digested with the restriction enzymes *Nhe*I-HF and *Bam*HI-HF and purified using the Wizard® SV Gel and PCR Clean-up System (Promega). Library generation in *S. cerevisiae* EBY100 cells was performed as described by Benatuil and colleagues (37). Each electroporation reaction consisted of 1.5 μg digested pCT plasmid and 4.5 μg insert. Altogether 10 transformation reactions per library were performed. Dilution plating was performed in order to determine the library size after three days.

### Yeast Library Screening

Yeast cells were cultured as described (38). In short, transformed yeast cultures were incubated over night at 30°C in SD-CAA medium. Cells were collected *via* centrifugation, resuspended in SG-CAA medium at OD_600_ = 0.7 and cultivated overnight at 20°C. For staining, cells were harvested and resuspended in BPBS (PBS + 0.1 % BSA). In general, 10^7^ cells were incubated in a volume of 20 µl and upscaled if necessary. For each staining a negative control in absence of target protein was included. For the first two sorting rounds cells were incubated with 1 µM SUP-B8 BCR and anti-c-myc-Biotin antibody (Miltenyi Biotec; 1:50 diluted) for 30 min on ice. After washing with BPBS, antigen binding was detected by application of anti-Human IgG Fc-PE conjugate (Affymetrix eBioscience; diluted 1:75) or Goat F(ab′)2 Anti-Human Lambda-PE (Southern Biotech; diluted 1:80) in combination with Streptavidin conjugated to APC or PE (Affymetrix eBioscience; diluted 1:75). Afterwards, cells were washed once with BPBS, resuspended in PBS and analyzed with a BD Influx™ cell sorter following the instructions of the manufacturer and analyzed via BD FACS™ Sortware v1.0. Setting of sort gates typically allowed 0.1% to 0.3% false-positive cells as judged by control staining in the absence of antigen. After sorting cells were subsequently transferred to SD-CAA medium for further incubation at 30°C for 2 days. Further sorting rounds were performed with at least a 10-fold excess of cells sorted out in the previous round to guarantee a sufficient coverage of the enriched populations. After enrichment of antigen-binding variants yeast cells plasmid DNA from antigen-binding populations was isolated using Zymoprep Yeast Plasmid Miniprep Kit (Zymo Research) and transformed into DH5α competent *E. coli* cells. After incubation overnight plasmids were isolated and sent out for sequencing utilizing pCT_seq_lo and pCT_seq_up oligonucleotides.

### Expression of Selected vNAR-Fc Variants

For expression of soluble vNAR molecules, genes were reformatted *via* fusion to human IgG1 Fc region encoded in the mammalian expression vector pEXPR-IBA42 (CosmoBio). For ligation *Nhe*I and *Apa*I sites and parts of the IgG1 hinge region were introduced *via* PCR. After restriction of entry plasmids and vNAR genes with the respective restriction enzymes and ligation and transformation of *E. coli*, sequencing of constructs was performed followed by transient transfection of Expi293F^TM^ cells using polyethyleneimine (Polysciences) according to the instructions of the manufacturer (Thermo Fisher Scientific). After 5 days, culture supernatants were harvested, sterile-filtered (0.22 μm Filtropur S 0.2, Sarstedt) and purified using Protein A spin columns. Buffer exchange was performed *via* dialysis against PBS using a membrane with a MWCO ~ 14 kDa.

### Binding Kinetics

For evaluation of binding kinetics parameters measurements were carried out on Octet RED96 system (FortéBio) at 30°C and 1000 rpm agitation in 200 µl. SUP-B8 BCR molecules were loaded on Anti-Human Fab-CH1 2nd Generation at 2 µg/mL in KB for 80 s. After that tips were transferred to kinetics buffer (KB; PBS, 0.1% Tween 20 and 1% BSA) for 60 s for sensor rinsing. Subsequently, association of varying concentrations from 2.4 to 200 nM of different vNAR-Fc variants were measured for 300 s followed by dissociation for 300 s in KB. In each experiment, a negative control was performed by incubating the captured BCR molecules with unrelated antigen or incubating tips loaded with unrelated antigen with soluble vNAR-Fc. Data fitting and analysis was conducted with FortéBio data analysis software 9.1 with a 1:1 Langmuir binding model after Savitzky–Golay filtering.

### Cellular Binding

Prior to analysis proteins were biotinylated using EZ-Link™ Sulfo-NHS-LC-Biotin (ThermoFisher) according to the instructions of the manufacturer. In scope of analyzing cellular binding, cells were sedimented and washed three times with 1% BPBS followed by incubation with the respective biotinylated vNAR-Fc constructs (250, 50 or 10 nM vNAR-Fc in 1% BPBS) for 1 h. In the next step cells were washed and incubated with fluorescently labeled Streptavidin-APC (diluted 1:75) for 20 min and washed once again. In the end, cells were analyzed using a BD Influx flow cytometry device.

### Generation of vNAR Antibody-Drug Conjugates

vNAR antibody-drug conjugates were generated upon enzymatic ligation of the antimitotic agent Monomethyl Auristatin E (MMAE) to the Fc fragment. Therefore, a genetically engineered *C*-terminal LPETGG extension was modified by an activity-optimized sortase variant (eSrtA) (39) from the *Gram*-positive bacterial strain *Staphylococcus aureus* as described elsewhere (40). Briefly, vNAR-Fc molecules were incubated in the presence of 0.1 equivalents sortase A and 10 equivalents GGG-vc-PABA-PEG_3_-MMAE in reaction buffer (150 mM NaCl, 50 mM TRIS, 10 mM CaCl_2_, pH 7.5 adjusted with HCl) for 1 h at 22°C. After protein modification, antibody-drug conjugates were purified *via* protein A chromatography (GE Healthcare), buffer-exchanged and concentrated to required concentrations (Amicon® Ultra 15 mL, Millipore).

### Cell-Based Assays

#### Isolation of Human Effector Cells

Human macrophages were generated by an adherence method utilizing monocyte-attachment medium (PromoCell, Heidelberg, DE). After an incubation for 24 h in serum-free X-Vivo medium (Lonza, Basel, CH), M-CSF (PeproTech, Rocky Hill, CA, USA) was added for macrophage generation. Human MNC were isolated from peripheral blood of healthy volunteers, as previously described (41) using Ficoll® (GE Healthcare, Chicago USA).

#### Chromium Release Assays

ADCC was analyzed in [51Cr] release assays as described previously (41). Briefly, effector cells, antibodies and medium were added to round-bottom microtiter plates (Nunc, Rochester, NY, USA) and the assay was started by adding target cells. After 3 h at 37°C, [51Cr] release from triplicate samples was measured. The percentage of cellular cytotoxicity was calculated using the formula: percentage of specific lysis = (experimental cpm − basal cpm)/(maximal cpm − basal cpm) × 100; maximal [51Cr] release was determined by adding 2 % v/v Triton-X-100 to target cells, and basal release measured in the absence of sensitizing antibodies and effector cells.

#### Phagocytosis Assays

ADCP was analyzed by high-throughput microscopy using the IncuCyte® (Sartorius, Göttingen, DE) system. Human macrophages served as effector cells in ADCP. For fluorescence staining, 1 × 10^6^ target cells were labeled with 0.5 µg/ml pHrodo (ThermoFisher, Waltham, MA) for 1 h at room temperature. Afterwards, target cells were washed two times in PBS and diluted in either cell culture medium or 0.1 M glycine pH 3 as positive control. The effector cell to target cell ratio (E:T ratio) was 1:1 with 0.4 × 10^5^ target cells as well as macrophages being seeded per well. After adding antibodies at various concentrations, ADCP was measured for 12 h at an interval of 30 min and analyzed using IncuCyte® software tools.

#### Internalization Assays

vNAR-Fc fusion proteins were conjugated with pHAb amine reactive dye according to manufacturer's recommended protocol (Promega). Briefly, antibodies were reacted at lysine amino acids with a 20 molar excess of amine reactive pHAb dye for 1 h. Unbound dye was subsequently removed using a Zeba desalting column (Thermo-Scientific). For internalization, B cells were plated at the density of 2 × 10^4^ vial cells per well and treated overnight with pHAb conjugated vNAR-Fc antibodies at desired concentrations. Plates were read on a fluorescent plate reader at Ex/Em: 532 nm/560 nm.

#### Cell Proliferation Assays

Cytotoxic effect of vNAR constructs was evaluated by exposing on-target SUP-B8 lymphoma B-cells or off-target cells to different antibody concentrations. Cell viability was analyzed 72 h after addition of vNAR-Fc variants with the CellTiter 96® AQueous One Solution Cell Proliferation Assay (Promega). Briefly, 1 × 10^4^ cells were seeded per well in a 96-well plate in 90 μl RPMI 1640 medium and treated with 10 μl vNAR-Fc antibody to reach final concentrations of ~20 pM up to 1 µM. After 72 h, 20 μl of the MTS solution were added per well and the plates were incubated for 2 h under standard conditions. Finally, the absorption of each well was measured at 490 nm in a Tecan reader. Cell proliferation in reference wells with untreated cells was set to 100 %.

### Statistical Analysis

Statistical analysis was conducted using SigmaPlot 12.5 (Systat Software, Inc.). Data are presented as mean ± standard error of the mean (SEM) of at least three independent experiments. Statistical significance was determined using a two-way ANOVA test (Bonferroni t-test). *p* values <0.05 were considered to be statistically significant.

## Results

### Identification and Generation of Lymphoma Cell-Specific B-Cell Receptors

In order to identify lymphoma BCRs and provide them in soluble form for library screening to obtain anti-idiotypic shark-derived antibodies, three different lymphoma cell lines, namely Daudi, IM-9, and SUP-B8 were used. First step was the generation of cell-specific soluble BCR molecules for their later use as antigen in a FACS-based selection process. Therefore, all cell lines were cultured, followed by RNA extraction and subsequent cDNA synthesis using random hexamer primers. Afterwards, the synthesized cDNA served as template for amplification of V_H_ and V_L_ genes using primers reported in a study by Maleshko et al. (34) that elucidated a specially designed panel of primers for the rapid amplification of variable regions of tumor immunoglobulins (Tab. S1). The primers enabled the specific amplification of four possible immunoglobulin chain types IgM, IgG, IgK, and IgL. After amplification of V_H_ and V_L_ domains showing the correct fragment size (**Figure S1A**), purified PCR products were sent for sequencing. Resulting sequences were aligned to the published sequences of all three BCR variants (42, 43) and can be found in **Figure S2**. To use the 3 BCRs as antigen in FACS based screening process in the next steps, the variable domains were cloned on the Fc-scaffold of matuzumab (44). For heavy chains, V_H_ domains of matuzumab were exchanged against the identified BCR V_H_ domains. Due to the lack of correct restriction sites for light chains, V_L_ of Daudi and IM-9 were fused via PCR to C_Kappa_ domains and V_L_ of SUP-B8 to C_Lambda_, respectively (**Figure S1A**).

After sequence verification, all constructs were cloned into pTT5 vectors for expression in HEK 293F cells as described elsewhere (45). Five days after transient transfection, cell cultures were harvested and supernatants were purified using Protein-A spin columns followed by dialysis against PBS. Analysis using SDS-PAGE revealed the presence of all three BCR variants after purification (**Figure S1B**). The BCRs of SUP-B8 and IM-9 showed the correct band pattern with heavy and light chain bearing the correct size. However, the BCR variant of the Daudi cell line showed a smearing band corresponding to the light chain with a higher apparent molecular size than predicted. We did not further investigate whether this difference in molecular weight is due to Daudi V_L_ domain glycosylation.

### Yeast Library Screening

**Figure 2:**
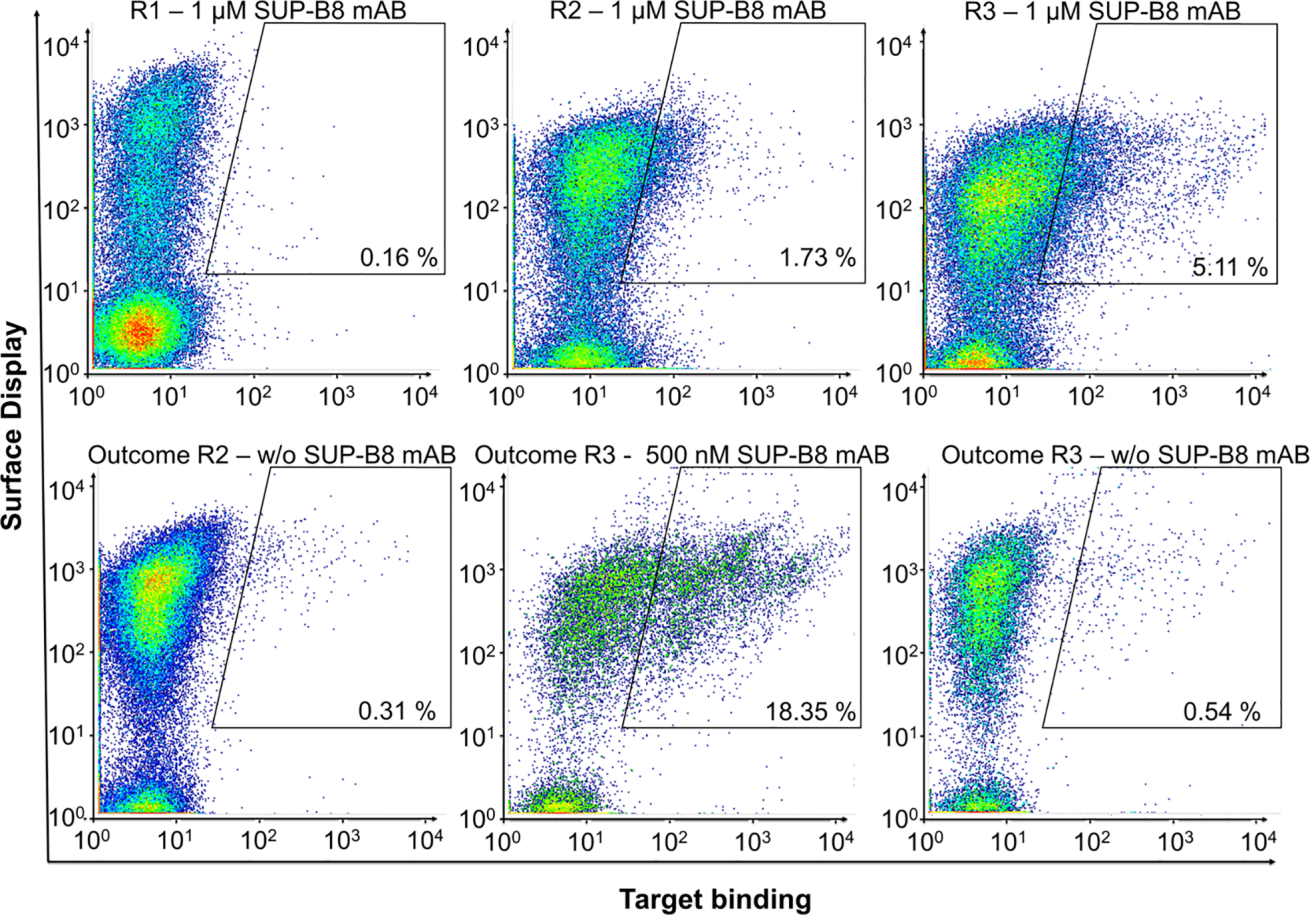
Shark-derived vNAR library screening against the BCR of cell line SUP-B8. Sorting gates, percentages of cells in the respective gate and target concentrations are shown. Anti-Human Fc-PE served as detection reagent. 1 d after induction, yeast cells were labeled for parallel detection of antigen-binding and surface presentation. In each round, cells in the sorting gate of the upper plots were isolated, grown and induced for the next round of selection. Outcome selected rounds of screening were analyzed for specific enrichment (lower plots).

To investigate whether specific anti-idiotypic vNARs can be obtained, the BCR of cell line SUP-B8 was utilized as antigen in a FACS-based screening process using the semi-synthetic vNAR yeast surface display library with randomized CDR3 loops in different lengths of 12 to 18 amino acids (32). Consequently, the libraries were screened by FACS for binders of recombinant SUP-B8 BCR protein. Target binding was identified by indirect antigen fluorescence staining. vNAR surface display level was analyzed simultaneously by labeling of the *C*-terminally located c-Myc tag. Negative controls in absence of antigen were carried out to evaluate the enrichment of binders directed against the staining reagents considered in the gate strategy. As depicted in **Figure 2**, we were able to enrich a SUP-B8 BCR-positive yeast population within three consecutive screening rounds by employing 1 µM antigen. Moreover, no significant binding against secondary detection reagents was observed.

Polyclonal anti-human IgG Fc-PE conjugate served as detection reagent in each round. However, to prevent the isolation of vNAR variants directed against this reagent, the third sorting round was repeated using anti-human Lambda-PE, recognizing the C_Lambda_ domain of SUP-B8 BCR (**Figure S3**). To assess the specificity of the enriched binding populations, target binding to IM-9 BCR and Cetuximab was analyzed and revealed no significant binding (**Figure S4**).

A total of 15 yeast clones were selected and analyzed for their target-specific binding to the SUP-B8 BCR using flow cytometry (**Figure S5**). This resulted in 13 clones showing surface presentation and specific BCR binding. Afterwards, sequencing was performed to evaluate the CDR3 loop sequences and to determine the clone diversity still present in this populations.

**Figure 3:**
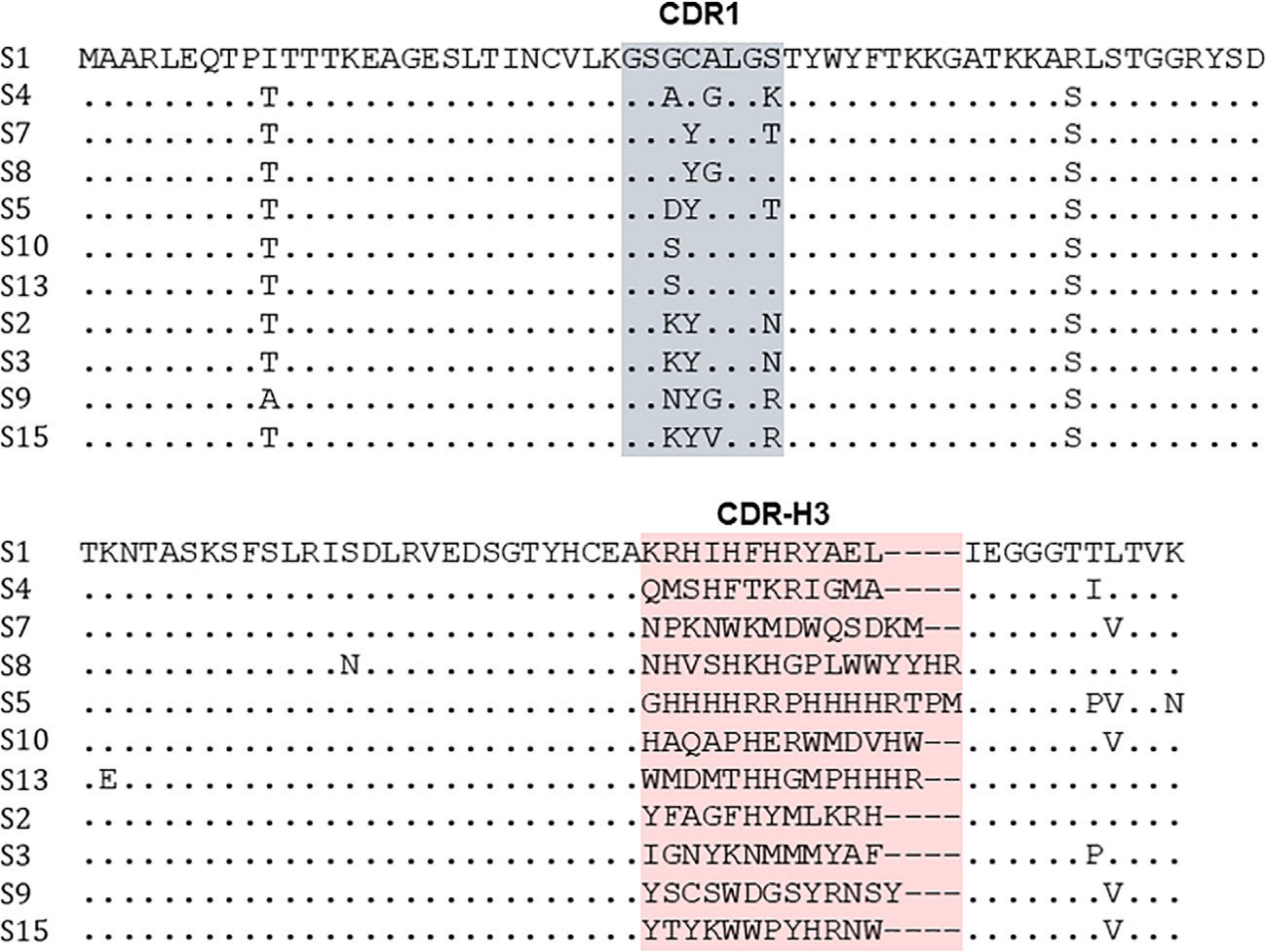
Sequence alignment of vNAR-Fc variants binding the BCR of lymphoma cell line SUP-B8. Sequencing was performed with clones derived from population after three rounds of FACS screening using yeast display. CDR1 and CDR3 binding sites are depicted in grey and red.

Sequencing revealed the presence of 11 unique single clones showing different CDR3 loop lengths of 12 to 16 amino acids (**Figure 3**). No variant comprised of 18 amino acids in CDR3 was enriched in the screening process.

### Generation and Characterization of vNAR-Fc Variants

For further characterization of the identified variants, eight clones in total were chosen for reformatting based on reliable sequence patterns and expressed as vNAR-Fc fusion proteins in HEK 293F cells. After production and purification *via* protein A affinity chromatography, the proteins were assessed by SDS-PAGE, indicating for five clones bands with the expected molecular weight of approximately 40 kDa as well as a high purity (**Figure S6**). For characterization, binding kinetics of binders resulting from screening campaigns were determined *via* biolayer interferometry (BLI). This resulted for four clones in binding affinities ranging from low single-digit nanomolar to low double-digit nanomolar binding constants (**Figure S7**).

**Figure 4:**
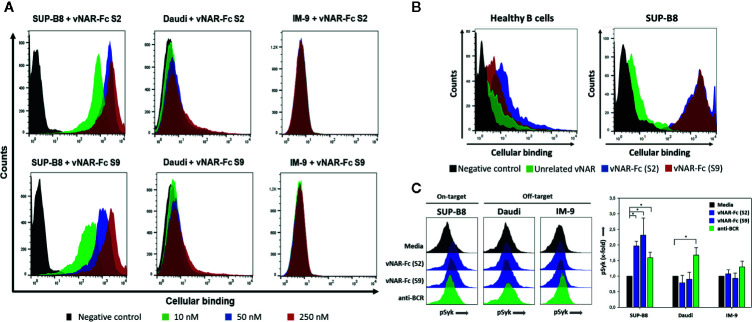
Specific cellular binding of vNARs to cell line SUP-B8. Two generated vNAR-Fc variants were analyzed for their binding to the B-cell lymphoma cell lines SUP-B8, Daudi and IM-9 by flow cytometry. 2 × 10^5^ cells were incubated with biotinylated vNAR-Fc molecules followed by incubation with streptavidin-APC. Black: only secondary reagent; red: 250 nM vNAR-Fc; blue: 50 nM vNAR-Fc; green: 10 nM vNAR-Fc **(A)**. CFSE-labeled SUP-B8 cells were mixed with B cells (ratio 1:1) isolated from healthy donors and treated with a saturating 100-nM concentration of biotinylated vNAR-Fc antibodies followed by staining with streptavidin-APC. Black: only secondary reagent; green: unrelated anti-matuzumab vNAR-Fc; blue: vNAR-Fc (S2); red: vNAR-Fc (S9) **(B)**. Lymphoma B cells were incubated with vNAR-Fc antibodies at a saturating 100-nM concentration. After incubation for 5 min at 37°C, cells were fixed with paraformaldehyde followed by permeabilization with ice-cold methanol. Intracellular Syk phosphorylation was detected upon staining with an anti-phospho Syk antibody (Cell Signaling Technology). Significant differences (*p* ≤ 0.05) between antibody and control treatment were determined using a one-way ANOVA test (Holm-Sidak test) and are depicted by * **(C)**. Results are shown as mean ± SD and are representative of three independent experiments.

To assess the specificity of soluble vNAR-Fc variants, binding to the BCRs of the unrelated cell line IM-9 was investigated. This resulted in three variants S2, S4, and S9 that revealed mainly specific binding whilst one variant (S7) showed significant binding intensity to the unrelated BCR of cell line IM-9 (**Figure S8**). Within this study we aimed to demonstrate the feasibility of shark-derived vNAR antibody fragments selected *via* yeast surface display for specific targeting of lymphoma cells. Cellular binding to SUP-B8 cells was therefore analyzed to prove the binding not only to the recombinantly expressed BCRs used as antigen for screening procedure but also to the native surface-presented BCR. Prior to analysis of cellular binding, surface expression of BCRs was verified by staining cells with anti-human lambda-PE or anti-human kappa-PE (**Figure S9**). Selective cellular binding was analyzed by comparing the binding to SUP-B8 cells with unspecific binding to BCR-presenting Daudi and IM-9 cells. Binding experiments indicated for 2 variants S2 and S9 a highly specific cellular binding in an idiotypic manner (**Figure 4A**). Moreover, experiments including primary B cells isolated from healthy donors demonstrated the capability of the vNAR-Fc fusion proteins to discriminate between the idiotype of the malignant clone and a plethora of idiotypes expressed by healthy B cells of an individual (**Figure 4B**). Of note, higher cell surface BCR expression levels were observed on lymphoma B cells compared with healthy B cells (**Figure S10**). Next, the ability of the vNAR-based antibodies to cluster the idiotype of the lymphoma B cells triggering BCR signal transduction was investigated. Antibody treatment of lymphoma cell lines showed stimulation of intracellular Syk phosphorylation, revealing specific mediation of BCR signaling (**Figure 4C**).

Given the final goal of specific opsonizing lymphoma B cells, the impact on receptor-mediated endocytosis was also investigated. vNAR-Fc antibodies revealed high internalization levels upon specific engagement of the BCR (**Figure S11**).

### vNAR-Mediated Tumor Cell Killing

Previous publications have shown the potential of BCR-binding peptibodies bearing four binding valences to kill tumor cells *in vitro* specifically by inducing cell apoptosis upon clustering of surface BCRs (23). Initial experiments utilizing both vNAR-Fc variants bearing only two binding valences showed no significant cell killing effect on SUP-B8 cells (**Figure S12A**). Likewise, a tetravalent version with four vNARs fused to the hinge region mediated no cell killing. To analyze whether the novel molecules trigger Fc-mediated effector functions, the capability of the vNAR-Fc molecules to specifically mediate ADCC and ADCP against malignant B-cells was investigated in ^51^chromium release assays using increasing antibody concentrations and PBMCs or macrophages, respectively, as effector cells (**Figure S13**). For unknown reasons, no antibody-dependent cell-mediated cytotoxicity or antibody-dependent cellular phagocytosis was observed in these assays.

**Figure 5:**
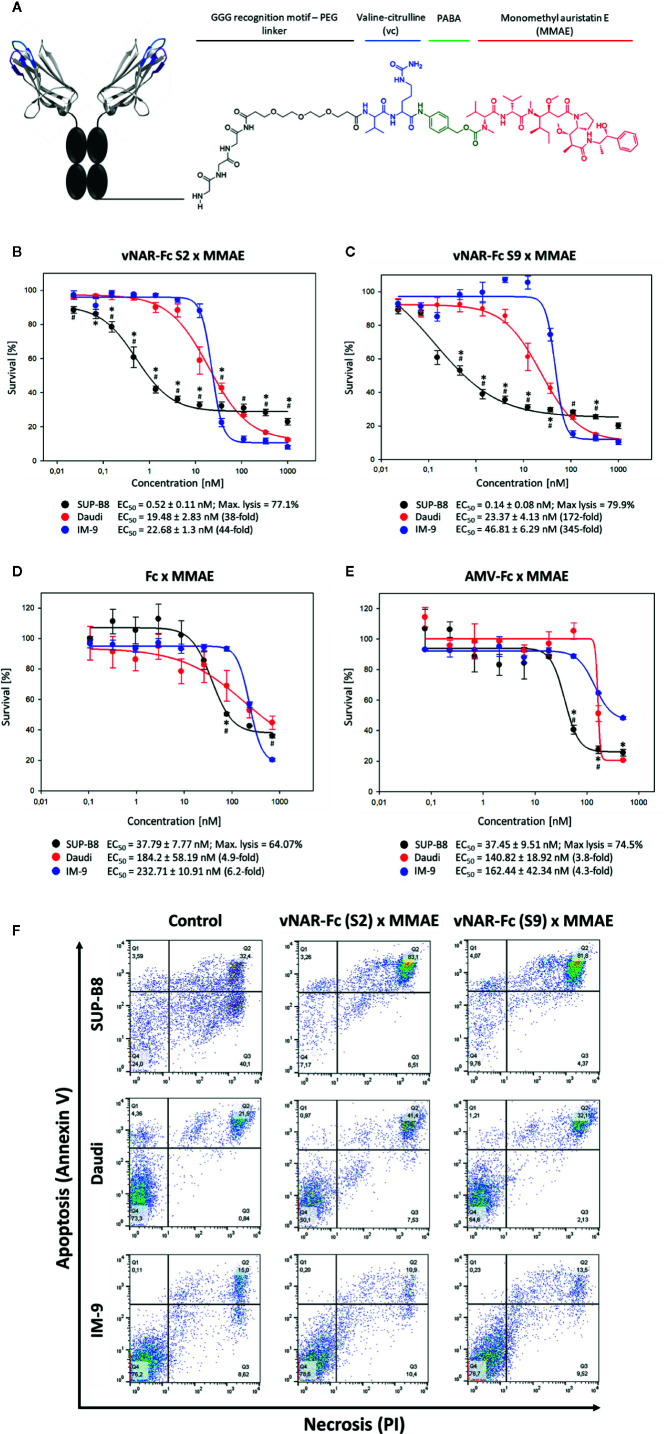
Induction of cytotoxicity in SUP-B8 cells by vNAR-based antibody-drug conjugates. A genetically engineered Fc fragment was MMAE-modified by using a sortase to generate vNAR-based antibody-drug conjugates. PABA: *p*-aminobenzoic acid **(A)**. On-target SUP-B8 as well as off-target Daudi and IM-9 wells were exposed to varying concentrations of two different MMAE-conjugated vNAR-Fc antibodies **(B, C)**. Fc-mediated cytotoxicity was assessed upon cell treatment with MMAE-modified solitary Fc fragment **(D)** and unrelated MMAE-conjugated anti-matuzumab vNAR-Fc (isotype control) **(E)**. vNAR-mediated cytotoxicity was measured based of cell proliferation after 72 h of antibody treatment. Results are shown as mean ± SEM and are representative of three independent experiments. Significant differences (*p* ≤ 0.05) between on-target and control Daudi and IM-9 cells were determined using a two-way ANOVA test (Bonferroni t-test) and are depicted by * and #, respectively. Mediation of apoptosis and necrosis was investigated upon treatment of 3 × 10^5^ cells with a 500-nM concentration of vNAR-ADCs. After 24 h of incubation, cells were stained with Annexin V and propidium iodide (PI) following manufacturer’s protocol (Rottitest® Annexin V, Carl Roth) and analyzed by FACS **(F)**.

As an alternative approach, we intended to generate antibody-drug conjugates for specific induction of cell killing in SUP-B8 lymphoma B-cells. Therefore, sortase A-mediated modification of our two most promising vNAR candidates S2 and S9 with monomethyl auristatin E (MMAE) was carried out (**Figure 5A**).

To this end, on-target SUP-B8 cells as well as off-target Daudi and IM-9 cells were treated with MMAE-conjugated vNAR-Fc constructs at concentrations ranging from ~20 pM to 1 µM. S2 and S9 antibody-drug conjugates mediated specific cytotoxicity in target SUP-B8 cells with IC_50_ values in picomolar range (517 nM and 136 nM, respectively) and similar maximal level of lymphoma cell lysis (**Figures 5B, C**). A cytotoxic effect was observed in off-target Daudi and IM-9 cells at higher antibody concentrations, showing, however, up to 345-fold higher EC_50_ values. Induction of specific cell death was additionally confirmed in an Annexin V FACS assay (**Figure 5F**). Of note, no significant killing effect could be detected on unrelated CHO cells (**Figure S12B**). Cell death induced in off-target cell lines might not be vNAR-related, as shown in experiments including isotype controls as well as vNAR-devoid Fc-MMAE molecules (**Figures 5D, E**). Differences observed between cell lines might be derived from varying overall sensitivity to the drug.

## Discussion

Using anti-idiotypic antibodies to target lymphoma B-cells has aroused interest in the scientific community a long time ago. The BCR might represent an ideal tumor-specific marker since this functionally active molecule is unique for each B-cell clonal population. Indeed, anti-idiotype antibody therapy in lymphoma patients has been shown to be successful in small clinical studies (46). The consequent need of developing a custom-tailored therapy, however, delayed further progress.

In this study we demonstrate the suitability of semisynthetic shark-derived vNAR antibodies for the development of a cost and time-effective personalized therapy against B-cell lymphoma. Thus, a yeast-displayed CDR3-randomized vNAR library generated from non-immunized bamboo sharks was used. First, immunoglobulin variable fragments of lymphoma cell lines were cloned, followed by reformatting and subsequent expression of recombinant idiotype proteins. A possible alternative to this procedure would have been the generation of hybridoma cell lines enabling the secretion of soluble BCR (24) which would have been a far more time-consuming process.

Furthermore, sequence analysis revealed for BCRs of cell lines Daudi and IM-9 comprising a kappa light chain whereas cell line SUP-B8 showed the sequence pattern of a lambda light chain. Consequently, the V_L_ domains were cloned on the respective constant domains to prevent the generation of molecules lacking stability (47). Cloning into expression vectors enabled the production as full-length IgG bearing an IgG1 Fc and the subsequent purification *via* protein A columns. BCRs of SUP-B8 and IM-9 showed the expected band patterns with heavy and light chains with correct sizes. However, analyzing Daudi BCRs revealed a light chain with a smearing band corresponding to the light chain with a higher apparent molecular size than expected. This might be caused by the presence of two motifs (N-X-S/T) for glycosylation sites in the CDR loops of Daudi V_L_ (48). In general, BCR CDR loops frequently acquire sequence motifs upon somatic mutation that serve as sites for N-glycosylation e.g. in follicular lymphoma (49).

Previous studies have shown the potential of vNARs to interact with the paratope of monoclonal antibodies in an anti-idiotypic manner (33). This might be caused by anti-idiotypic vNARs being structurally capable of engaging interspaces at the interaction site of heavy and light chains of a monoclonal antibody, which could be attributed to their elongated CDR3 binding sites (27). After FACS-based yeast surface display screening of the semi-synthetic vNAR-library, the resulting population of binders still showed a high diversity which enabled to select the most promising variants. Although no counter-screen using unrelated antibodies was implemented, it could be shown that the majority of binders specifically recognize the antigen in an idiotypic manner. Furthermore, it has to be mentioned that all binders resulting from this study mainly bind *via* amino acid residues in the CDR3. Eight vNAR variants were reformatted via fusion to a human Fc. Of these, five were able to be expressed at reasonable yields (50–100 mg/L). Depending on CDR sequences, it is a well-known problem of many single-chain antibodies to show low expression yields (50, 51). Evaluation of vNAR affinities was performed by application of biolayer interferometry revealing binding constants in the single-digit nanomolar range. Specificity was assessed again with soluble protein which resulted for three variants (S2, S4 and S9) in highly specific binding to the BCR of cell line SUP-B8. This was also true for binding to BCR presenting SUP-B8 cells clearly showing a specific cellular binding. This also indicated the recombinantly expressed BCR being folded similarly as the wildtype membrane-anchored BCR. The combinatorial diversity of the naïve antibody repertoire in humans is estimated to be at least 10^12^ unique BCRs (52). The immense plethora of different idiotypes within an individual could diminish binding thus limiting the effectiveness of the antibodies. In this study, vNAR-Fc fusion proteins were ultimately demonstrated to specifically engage the idiotype of a malignant cell population being able to distinguish from BCRs expressed by healthy B cells.

Receptor-mediated endocytosis plays an important role in cell death induction. BCR clustering and thus triggering of cell apoptosis might be impaired by rapid antibody internalization, while efficacy of antibody-drug conjugates is to a large extent driven by receptor-mediated antibody endocytosis followed by release of the toxic compound finally causing cell death (53). Treatment of lymphoma B cells with vNAR-Fc fusion molecules revealed BCR signal transduction as well as extensive internalization upon binding of the specific BCR. As expected, control lymphoma cell lines Daudi and IM-9 bearing different idiotypes showed significantly lower antibody internalization levels, likely due to Fc/Fcγ receptor interactions rather than upon unspecific vNAR-mediated BCR engagement. Torchia et al. have reported anti-idiotype peptibodies bearing four binding valences to kill lymphoma cells *in vitro* with specificity *via* induction of cell apoptosis by clustering the surface BCRs (23). Consequently, we investigated our most promising vNAR-Fc variants S2 and S9 towards their potential cell killing effect on SUP-B8 cells. This resulted in no observable cytotoxicity even at higher triple-digit nanomolar concentrations. The lack of vNAR-Fc-triggered BCR clustering is concordant with different reports describing that constructs with only two binding valences show significantly reduced receptor clustering (54). Moreover, neither addition of an anti-human Fc antibody to SUP-B8-bound vNAR-Fc nor application of a tetravalent construct with two additional *C*-terminally fused vNAR domains lead to a significant induction of apoptosis (data not shown). It is tempting to speculate that these observations are mainly reasoned through rapid internalization and degradation of vNAR-Fc/BCR complexes triggered by either BCR cross-linking or interaction of Fc region of vNAR constructs with the Fcγ receptors expressed on lymphoma B-cells (55). Upcoming studies may reveal whether this issue can be circumvented by cloning the genes coding for BCR-binding vNARs into higher multivalent antibody formats. In the case of VHHs, Drabak et al. reported that it is feasible to generate tetravalent VHH-Fc molecules either consisting of two VHH domains N-terminally fused to the constant domain or located at both termini of the Fc portion (56). Furthermore, it would be possible to use one of the several published scaffolds that enable multivalent conjugation of proteins *e.g.* verotoxin B (57, 58) or the C4-binding protein (59). Previous studies have shown that B cell activation is triggered by BCR-antigen engagement (60). Forthcoming studies might reveal more details regarding BCR activation and eventually internalization upon engagement of anti-idiotypic antibodies.

Next, we investigated the potential of the vNAR-Fc molecules to specifically mediate ADCC and ADCP against lymphoma B-cells. Unexpectedly, experiments revealed no activity to activate human PBMCs and macrophages for ADCC/ADCP. It is known that Fc glycosylation plays a critical role in binding to human Fc receptors and consequently in triggering ADCC or ADCP. Improper glycosylation patterns present on the vNAR-Fc antibodies might explain the inefficacy of the Fc fragment to induce antibody-mediated cytotoxicity/phagocytosis against malignant B-cells. In addition, a variety of target antigen characteristics, such as epitope location, antigen density or molecule design have been described to critically impact Fc-mediated effector functions. We did not further investigate these parameters within the scope of this work.

Besides triggering apoptosis by BCR clustering and engagement of effector cells, it is feasible to investigate the killing effect of an antibody-drug conjugate. Here, we report a patient-tailored approach in a proof of concept study based on the generation of vNAR antibody-drug conjugates that kill lymphoma B-cells upon specific vNAR-mediated targeting of the BCR. Enzymatic MMAE-modification of the SUP-B8 BCR-binding vNAR-Fc candidates S2 and S9 resulted in specific induction of cytotoxicity in on-target lymphoma B cells at picomolar concentrations. In contrast, the vNAR-ADCs showed no significant cytotoxic effect in unrelated CHO cells as well as off-target B cells in the same picomolar range. Of note, increasing ADC concentrations led to killing of off-target lymphoma B cells. Nonspecific cell death caused by lymphoma B-cell targeting antibody-drug conjugates has been observed in similar studies (61). As discussed before, this effect might be derived from antibody/antigen complex uptake and internalization *via* interaction with Fcγ receptors present on B cells. Experiments including vNAR-unrelated constructs as isotype control as well as solitary MMAE-modified Fc molecules support our hypothesis of Fc/Fcγ receptor interactions being responsible for unspecific cell killing induced in off-target Daudi and IM-9 cells. Furthermore, similar cell surface BCR expression levels were observed for all three lymphoma cell lines. Interestingly, we noticed that SUP-B8 cells respond more sensitive to both isotype control and MMAE-conjugated Fc domain. This might be explained by a higher surface Fcγ receptor density compared to Daudi and IM-9 cells, by an increased antibody/receptor complex internalization rate or by a different intracellular processing after antibody-toxin uptake. Despite inducing a nonspecific cytotoxic effect, the Fc region is responsible for improved stability and pharmacokinetics and may promote cell apoptosis by BCR clustering in toxin-free multivalent antibody formats.

ADCs for treatment of B-cell non-Hodgkin’s lymphoma and follicular or diffuse large B-cell lymphoma developed by Genentech/Roche are being investigated in actual Phase I and Phase II clinical trials (11). Currently, patients with B-cell lymphomas such as Burkitt lymphoma and several other Non-Hodgkin lymphoma types are treated with a combination of extensive chemotherapy with Rituximab that binds selectively the B-lymphocyte antigen CD20. Although this therapy has shown to be very effective, especially utilizing Rituximab for treatment has several potential disadvantages. On the one hand Rituximab opsonizes all cells that express the tumor-associated antigen CD20. The resulting suppression of the whole immune system increases the risk of infections and viral reactivation (12). On the other hand it also removes a large population of antigen-presenting cells (APCs) important for initiation of T-cell-mediated cellular immunity (62). Further risks are associated with loss of CD20 expression or the development of lymphoma cells showing mutated CD20 protein on their surface. As a consequence, cancer cells are not able anymore to be targeted by Rituximab (63, 64). Thus, incomplete depletion of malignant B cells often results in disease relapse and treatment resistance in patients (65).

Due to their remarkably attributes, harnessing heavy-chain only antibodies from the adaptive immune system of cartilaginous fish seems to be a promising approach for the development of novel therapeutic molecules. Given the substantial evolutionary distance of cartilaginous fish to human, however, potential immunogenicity should be taken into consideration. Yet, previous studies have demonstrated the feasibility of humanizing vNAR domains in order to prevent xeno-reactivity (66).

In summary, considering the BCR as tumor-specific cell surface marker which is also distinct from the BCRs displayed on non-malignant B cells, we developed a novel strategy for a custom-tailored targeted therapy to induce death in lymphoma B cells in a time efficient and inexpensive manner. The herein demonstrated versatile approach enables the generation of vNAR antibody drug conjugates against patient-specific lymphoma cells in a time of less than six months from time point of starting with material of lymphoma patients. The high specificity of our vNAR antibodies offers a therapeutic window enabling the eradication of lymphoma B cells without inducing overall immunosuppression derived from the ablation of the whole B-cell subset in patients. Future experiments may enable the generation of vNAR-Fc antibody formats with higher binding valences that allow for apoptosis induction of lymphoma B cells by BCR clustering.

## Concluding Remarks

Several therapies based on monoclonal antibodies as well as small molecules against malignant B cells have been developed during the last years. These treatments, however, target B cells in a non-specific manner, thus leading to immunosuppression and finally to severe side effects (12). The use of antibody-drug conjugates based on anti-idiotypic vNARs specifically targeting the BCR might represent a beneficial strategy to induce killing in lymphoma B cells. The versatile approach described here as an *in vitro* proof of concept may overcome technical hurdles derived from the development of a patient-tailored treatment against lymphoma-related malignancies.

## Data Availability Statement

The original contributions presented in the study are included in the article/**Supplementary Material**. Further inquiries can be directed to the corresponding author.

## Ethics Statement

The studies involving human blood donors were reviewed and approved by the ethics committee of the medical faculty of the Christian-Albrechts-Universität zu Kiel (Arnold-Heller-Str: 3;24105 Kiel).

## Author Contributions

AM, JG, and HK conceived and designed the experiments. AM, JG, LD, TR, and MP performed the experiments. AM, JG, TR, MP, and HK analyzed the data. SK and SZ gave scientific advice. AM, JG, TR and MP, and HK wrote the paper. All authors contributed to the article and approved the submitted version.

## Funding

This work was supported by the Department of Protein Engineering and Antibody Technologies at Merck KGaA, Darmstadt and by the Sektion für Stammzell- und Immuntherapie, Universitätsklinikum Schleswig-Holstein, Kiel. This work was funded in part by Deutsche Forschungsgemeinschaft through grant KO 1390/14-1. The funder was not involved in the study design, collection, analysis, interpretation of data, the writing of this article or the decision to submit it for publication.

## Conflict of Interest

The authors declare that the research was conducted in the absence of any commercial or financial relationships that could be construed as a potential conflict of interest.

## Abbreviations

ADC, antibody-drug conjugate; ADCC, antibody-dependent cellular cytotoxicity; ADCP, antibody-dependent cellular phagocytosis; Aga2p, *a*-agglutinin II subunit; APC, antigen-presenting cell; BCR, B-cell receptor; BLI, biolayer interferometry; BSA, bovine serum albumin; ELISA, enzyme-linked immunosorbent assay; CDR, complementarity-determining region; Fab, antigen-binding fragment; FACS, fluorescence-activated cell sorting; GPI, glycosylphosphatidyl-inositol; Id, idiotype; Ig, immunoglobulin; KB, kinetics buffer; mAb, monoclonal antibodies; MMAE, monomethyl auristatin E; PBMCs, peripheral blood mononuclear cells; PBS, phosphate-buffered saline; PCR, polymerase chain reaction.
