# Supplementary material for: Specific Targeting of Lymphoma Cells Using Semisynthetic Anti-Idiotype Shark Antibodies
Source: Front Immunol. 2020 Nov 26;11:560244. doi: 10.3389/fimmu.2020.560244 (PMC7726437; doi:10.3389/fimmu.2020.560244)
Supplement: Supplementary file 1 [file DataSheet_1.pdf]

Supplementary Material

1 Supplementary Figures

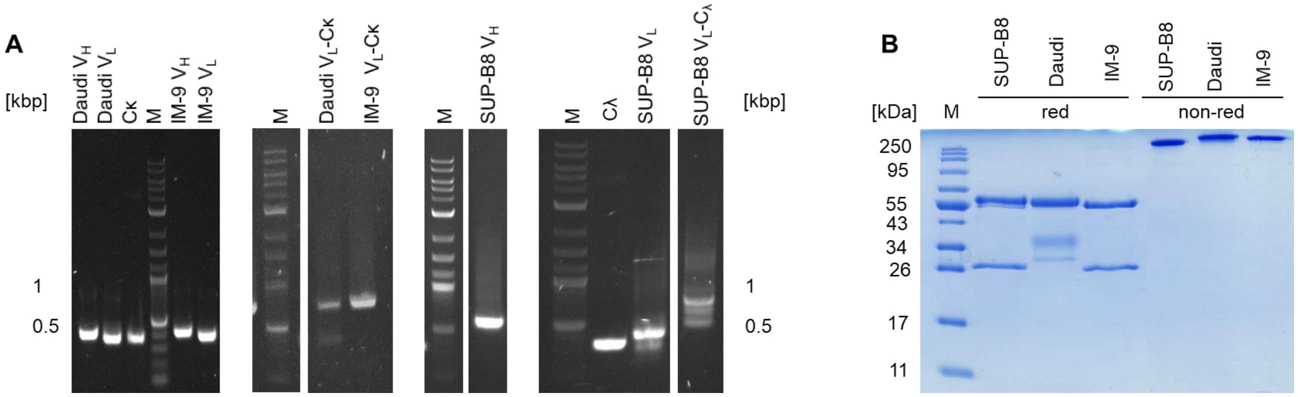

SUPPLEMENTARY FIGURE S1 | Generation of B-cell lymphoma-derived soluble BCR protein. BCR-coding genes were amplified separately using different primers binding in the FR1 and C_L/C_{H1} regions. For heavy chains, V_H domains of matuzumab were exchanged for the identified BCR V_H domains. For light chains, V_L of Daudi and IM-9 were fused *via* PCR to C_{Kappa} domains, V_L of SUP-B8 to C_{Lambda}, respectively **(A)**. Reducing SDS-PAGE of BCRs after expression in HEK 293F cells **(B)**. Using matuzumab as a scaffold, the respective V_H and V_L domains were exchanged for the identified BCR-derived variable domains. Purification was performed using Protein A spin columns. Molecular weight of heavy chains is approximately 55 kDa, of light chains ~26 kDa, respectively.

SUP-B8 Heavy Chain

```
                                            CDR-H1
published   QIQLVQSGGEVKKPGASVRVSCKASGYTFHSYGITWVRQAPGQGLEWMG
VH1         ...LVQSGGEVKKPGASVKVSCKASGYTFHSYGITWVRQAPGQGLEWMG
VH2         ...................VSCKASGYTFHSYGITWVRQAPGQGLEWMG
VH5         ..QLVQSGGEVKKPGASVKVSCKASGYTFHSYGITWVRQAPGQGLEWMG
                 CDR-H2
published   WISGYNGNTNYAQKLQDRVTMTTDTSTNTVYMEVRSLRSDDTAVYYCAR
VH1         WINGYNGYTNYAQKLQDRVTMTTNTSTNTVYMEVRSLRSDDTAVYYCAR
VH2         WINGYNGYTNYAQKLQDRVTMTTNTSTNTVYMEVRSLRSDDTAVYYCAR
VH5         WINGYNGYTNYAQKLQDRVXMTTNTSTNTVYMEVRSLRSDDTAVYYCAR
                 CDR-H3
published   DDCSGDNCYMSAYWGQGTLVTVSS
VH1         DDCSGDNCYMSAYWGQGTVVTVSS
VH2         DDCSGDNCYMSAYWGQGTVVTXSX
VH5         ........................
```

SUP-B8 Light Chain

```
                                           CDR-L1                        CDR-L2
published     QSVLTQPPSASGTPGQRVTISCSGSSSKIASNYVYWYQQVPGTAPKLLIYRDNQRPSGV
IgL_36-47     QSVLTQPPSASGTPGQRVTISCSGSSSNIASNYVYWYQQLPGMAPKLLIYRDNQRPSGV
IgL_51        QSVLTQPPSASGTPGQRVTISCSGSSSNIASNYVYWYQQLPGMAPKLLIYRDNQRPSGV
                                                   CDR-L3
published     PDRFSGSRSGTSASLAISGLRSDDEADYYCATWDDSLSGWVFGGGTKLTVL
IgL_36-47     PDRFSGSRSGTSASLAISGLRSDDEADYYCATWDDSLSGWVFGGGTKLTVL
IgL_51        PDRFSGSRSGTSASLAISGLRSDDEADYYCATWDDSLSGWVFGGGTKLTVL
```

IM-9 Heavy Chain

```
                                                       CDR-H1
published     LEVQLVESGGGLLQPGRALRLSCAASGFRFDDYAMHWVRQTPGKGLEWVA
VH3_rev       .....VESGGGLLQPGRALRLSCAASGFRFDDYAMHWVRQTPGKGLEWVA
VH1_rev       .....VQSGGGLLQPGRALRLSCAASGFRFDDYAMHWVRQTPGKGLEWVA
VH1_fwd_up    ............................RFDDYAMHWVRQTPGKGLEWVA
3_fwd_up      ............................RFDDYAMHWVRQTPGKGLEWVA
5_fwd_up      .............................FDDYAMHWVRQTPGKGLEWVA
                   CDR-H2
published     GISWNSDTIDYADSVKGRFTISRDNAKNSLYLQMNSLRAEDTALYYCTKE
VH3_rev       GISWNSDTIDYADSVKGRFTISRDNAKNSLYLQMNSLRAEDTALYYCTKR
VH1_rev       GISWNSDTIDYADSVKGRFTISRDNAKNSLYLQMNSLRAEDTALYYCTKR
VH1_fwd_up    GISWNSDTIDYADSVKGRFTISRDNAKNSLYLQMNSLRAEDTALYYCTKR
3_fwd_up      GISWNSDTIDYADSVKGRFTISRDNAKNSLYLQMNSLRAEDTALYYCTKR
5_fwd_up      GISWNSDTIDYADSVKGRFTISRDNAKNSLYLQMNSLRAEDTALYYCTKR
              CDR-H3
published     GGVTDIDPFDIWGQGTMVIVSS
VH3_rev       RGVTDIDPF.............
VH1_rev       RGVTDIDPF.............
VH1_fwd_up    RGVTDIDPFDIWGQGTMVIVSS
3_fwd_up      RGVTDIDPFDIWGQGTMVIVSS
5_fwd_up      RGVTDIDPFDIWGQGTMVIVSS
```

IM-9 Light Chain

```
                                            CDR-L1                        CDR-L2
published     ELQMTQSPSTLSASVGDRVTITCRASQSISPWLPWYQQKPGKAPKLLIYKASSLESGVPS
Vk2_lo        ....TQSPSTLSASVGDRVTITCRASQSISAWLAWYQQKPGKAPKLLIYKASSLESGVPS
Vk1_up        ...........................SISAWLAWYQQKPGKAPKLLIYKASSLESGVPS
                                                 CDR-L3
published     RFSGSGSGTEFTLTITSLQPDDFATYFCQHYNRPWTFGQGTKVEIKR
Vk2_lo        RFSGSGSGTEFTLTITSLQPDDFATYFCQHYNRPWT...........
Vk1_up        RFSGSGSGTEFTLTITSLQPDDFATYFCQHYNRPWTFGQGTKVEIKR
```

Daudi Heavy Chain

```
                                                CDR-H1
published       LEVQLVESGGNLVQPGGSLRLSCEVSGFSITSYGIHWVRQAPGKGLVWVS
VH3             LEVQLVESGGDLVQPGGSLRLSCEASGFTITSYGMHWVRQAPGKGLVWVS
VH5_fwd         ...QLVESGGDLVQPGGSLRLSCEASGFTITSYGMHWVRQAPGKGLVWVS
VH5_rev         ..................................................

                      CDR-H2
published       ETDNDGRDATYADSVKGRFTSLPDRANNTLYLQMNSLRVDDTAVYYCVRG
VH3             EIDNDGRDATYADSVKGRFTSLPDRANNTLYLQMNSLRVDDTAVYYCVRG
VH5_fwd         EIDNDGRDATYADSVKGRFTSLPDRANNTLYLQMNSLRVDDTAVYYCVRG
VH5_rev         .......DATYADSVKGRFTSLPDRANNTLYLQMNSLRVDDTAVYYCVRG

                  CDR-H3
published       NGQKCFDYWGQGTLVTVSS
VH3             NGQKCFDYWGQGTLVTVSS
VH5_fwd         NGQKCFDYW..........
VH5_rev         NGQKCFDYWGQGTLVTVSS
```

Daudi Light Chain

```
                                           CDR-L1                     CDR-L2
published       ELQMTQSPSSLSASVGDRVTITCRAGHNITNFLSWYQQKPGKAPTLLIYAVSNLQDGV
Vk1_up          ....TQSPSSLSACVGDRVTITCRAGHNITNFLSWYQQKPGKAPTLLIYAVSNLQRGV
Vk1_lo          ....TQSPSSLSACVGDRVTITCRAGHNITNFLSWYQQKPGKAPTLLIYAVSNLQRGV
Vk2_up          ..................VTITCRAGHNITNFLSWYQQKPGKAPTLLIYAVSNLQRGV

                                                  CDR-L3
published       PSRFSGSGSGAEFTLTISSLQPEDFATYYCQQNYNFSFTFGGGTKVDNKR
Vk1_up          PSRFSGSGSGAEFTLTISSLQPEDFATYYCQQNYNFSFTFGGGTKVDNKR
Vk1_lo          PSRFSGSGSGAEFTLTISSLQPEDFATYYCQQNYNFSFTFGGG.
Vk2_up          PSRFSGSGSGAEFTLTISSLQPEDFATYYCQQNYNFSFTFGGGTKVDNKR
```

SUPPLEMENTARY FIGURE S2 | Sequence overview over 3 BCRs from 3 different lymphoma cell lines Daudi, IM-9 and SUP-B8. After RNA extraction and cDNA synthesis BCR-coding genes were amplified separately using different primers binding in the FR1 and CL/CH1 regions. The resulting sequences were aligned to the published sequences, variations are depicted in red. The CDR boundaries are indicated above the sequence alignment.

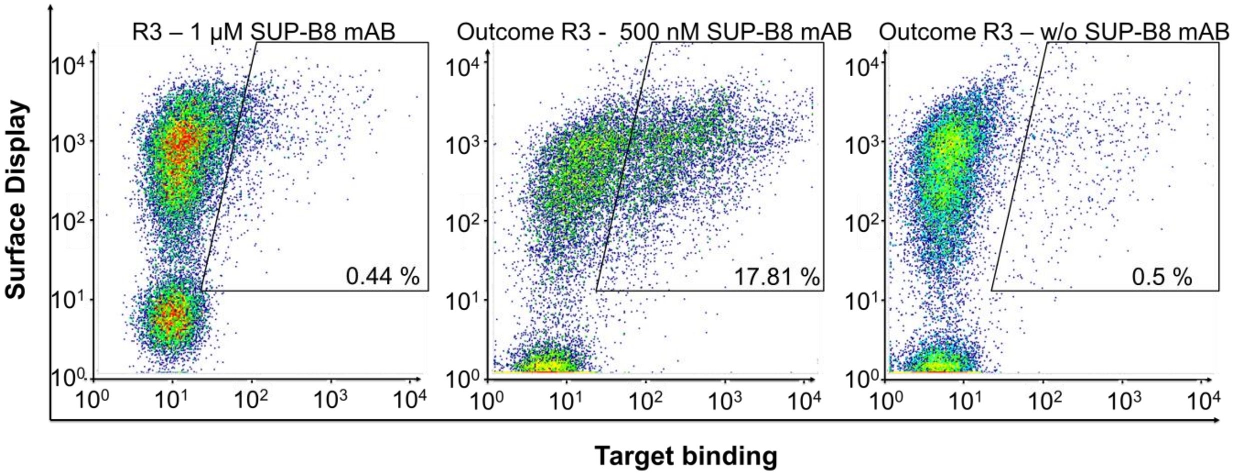

SUPPLEMENTARY FIGURE S3 | Shark-derived vNAR library screening against the BCR of cell line SUP-B8. In addition to the third screening round stained with anti-Human Fc-PE, the third round of screening was performed in parallel with antigen stained with anti-Human Lambda-PE. Sorting gates, percentages of cells in the respective gate and target concentrations are shown. One day after induction, yeast cells were labeled for parallel detection of antigen-binding and surface presentation. After screening cells in the sorting gate were isolated, grown and induced for the next round of selection.

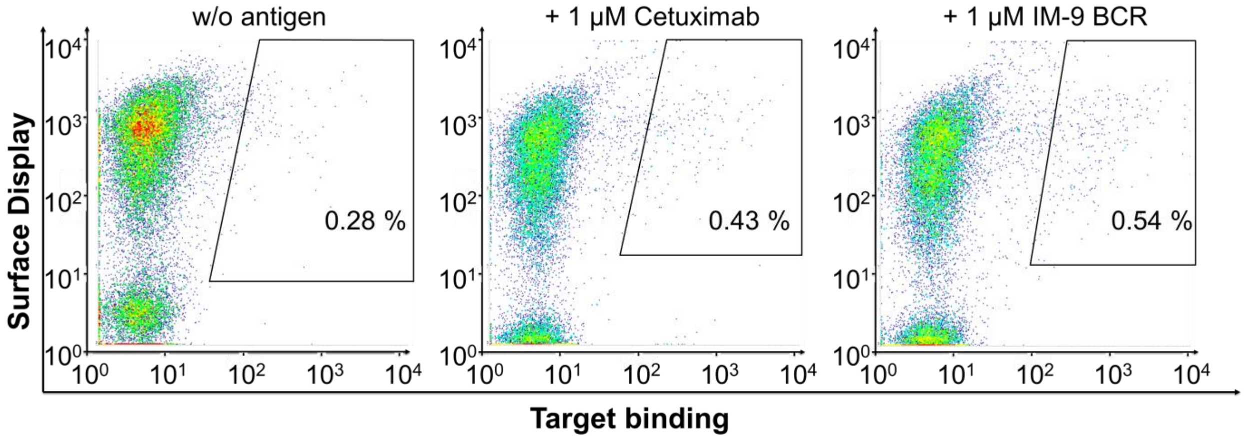

SUPPLEMENTARY FIGURE S4 | Specificity of vNAR-presenting yeast population after two rounds of sorting for SUP-B8-BCR binding variants as determined via binding assays on the yeast surface. A negative control in absence of antigen was performed whilst off-target binding was validated against the unrelated antibody cetuximab and the BCR of cell line IM-9. Surface presentation was detected by utilizing anti-myc biotin and SAPC, target binding was analyzed by using anti-human-FC PE conjugate. The percentage of cells localized in gate is depicted on each plot.

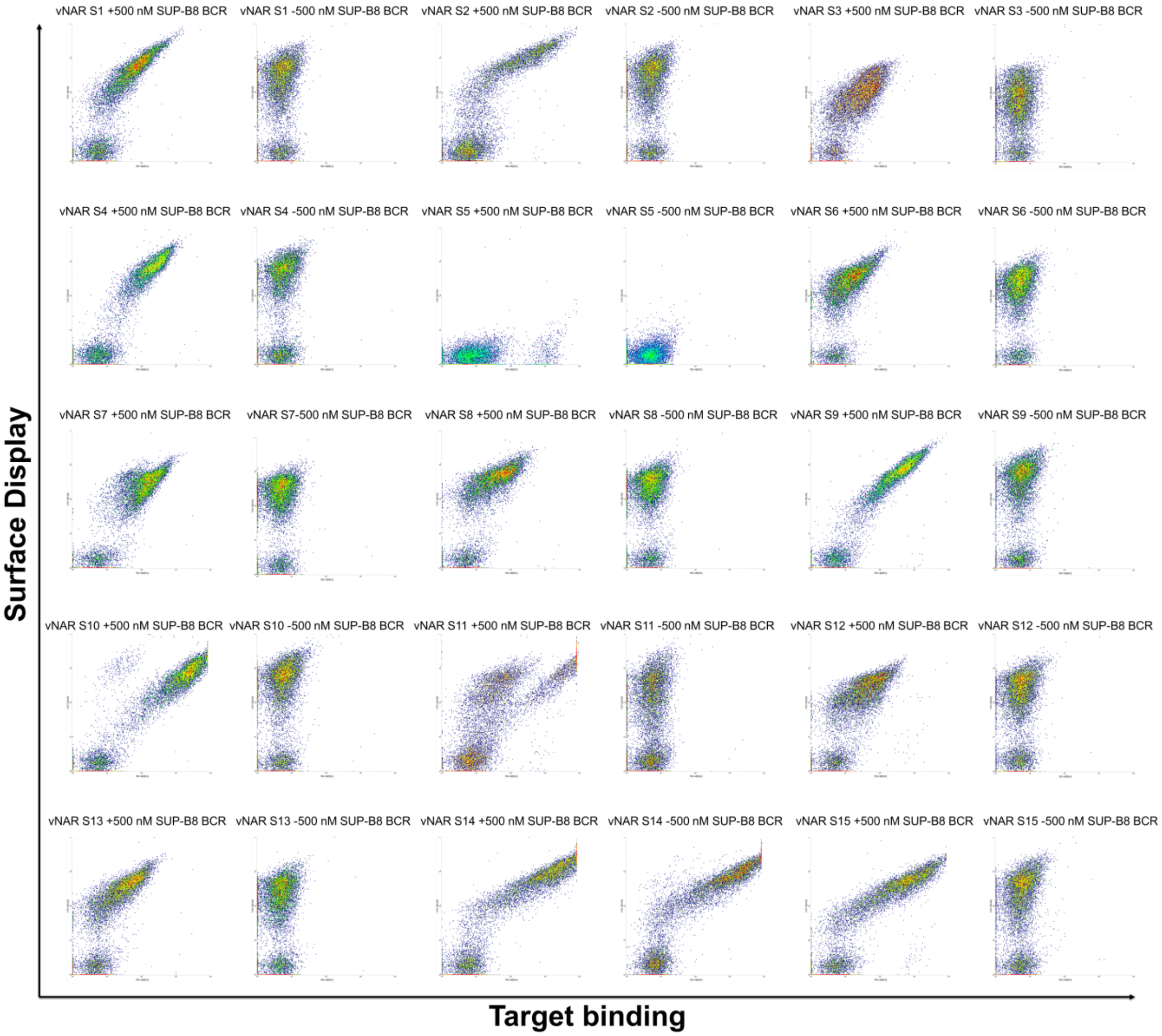

SUPPLEMENTARY FIGURE S5 | Single clone analysis after three rounds of sorting for SUP-B8 binding vNARs. Each respective single clone was measured after one day of induction. Cells were incubated with 500 nM SUP-B8 BCR, anti-human-Fc-PE conjugate served as detection antibody. For each clone a negative control in absence of antigen was performed.

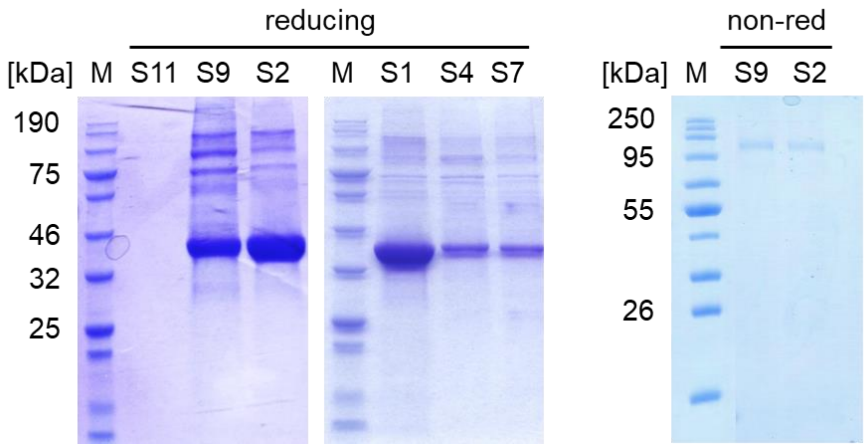

SUPPLEMENTARY FIGURE S6 | SDS-PAGE under reducing and non-reducing conditions of six vNAR-Fc constructs after Expression in HEK293 Expi cells and protein A purification. Molecular weight of vNAR-Fc molecules is approximately 80 kDa.

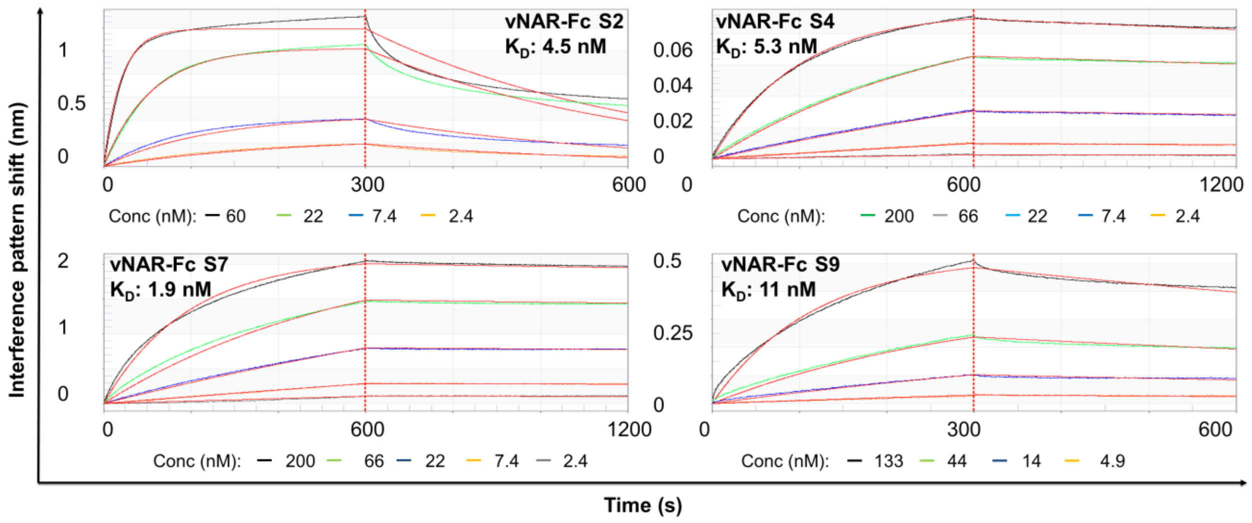

SUPPLEMENTARY FIGURE S7 | Binding kinetics of vNAR-Fc variants directed against the BCR of the SUP-B8 cell line as determined using Bio-Layer Interferometry and an Octet® RED96 system. BCR molecules were immobilized onto anti-human Fab-CH1 2nd Generation sensor tips. Association with varying concentrations of vNAR-Fc was measured for 300 or 600 s followed by dissociation measurement for 300 or 600 s. Fitting (red lines) of binding curves (colored lines) was calculated using a 1:1 binding model and Savitzky-Golay filtering. Target protein concentrations for each kinetic measurement and the resulting binding constants are depicted in each plot.

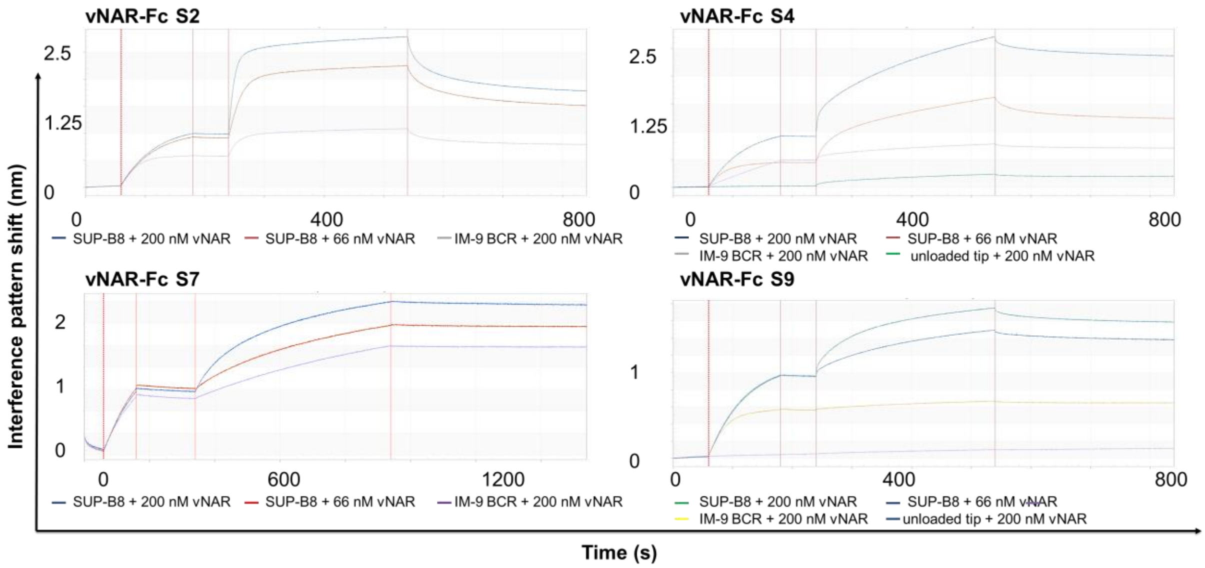

SUPPLEMENTARY FIGURE S8 | Raw Data of kinetics measurements of four vNAR-Fc variants S2, S4, S7 and S9 via biolayer interferometry with an Octet® RED96 system. BCR molecules were immobilized onto Anti-Human Fab-CH1 2nd Generation sensor tips. Association with varying concentrations of vNAR-Fc was measured for 300 or 600 s followed by dissociation measurement for 300 or 600 s. In each plot a control measurement with IM-9 BCR immobilized to the tips was implemented. Target protein concentrations for each kinetic measurement are depicted in each plot.

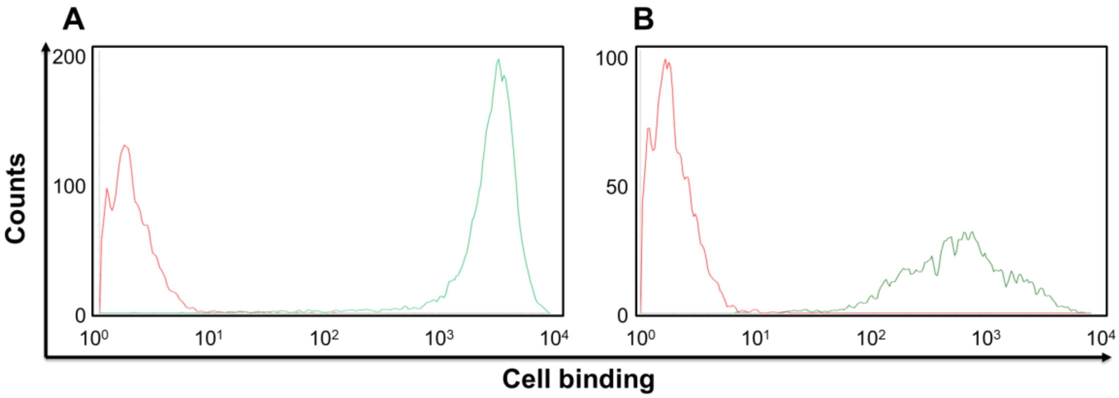

SUPPLEMENTARY FIGURE S9 | Confirmation of BCR surface expression of cell lines SUP-B8 (A) and IM-9 (B). Before measurement, cells were treated according to section 2.1. Red: unstained cells; green: anti-lambda-PE conjugate (for SUP-B8 cells) and anti-kappa-PE conjugate (for IM-9 cells). Cells were incubated 30 min on ice with the respective detection antibody.

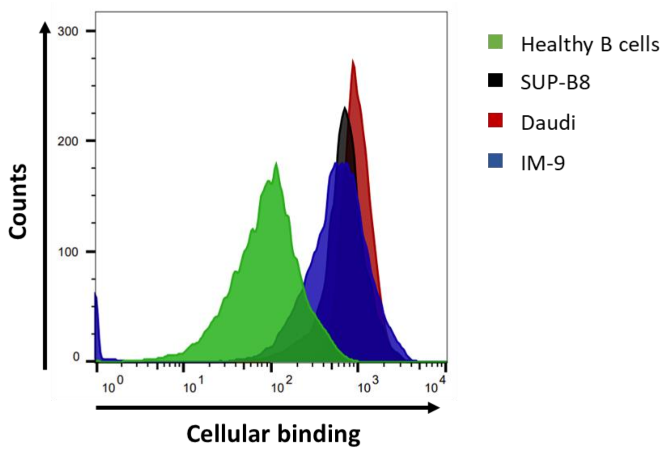

SUPPLEMENTARY FIGURE S10 | Comparison of cell surface BCR expression levels on lymphoma B cells and primary B cells isolated from healthy donors. Before analysis, cells were incubated 30 min on ice with anti-lambda-PE and anti-kappa-PE conjugates. Green: healthy B cells; black: SUP-B8; red: Daudi; blue: IM-9. Results are representative of 3 independent experiments.

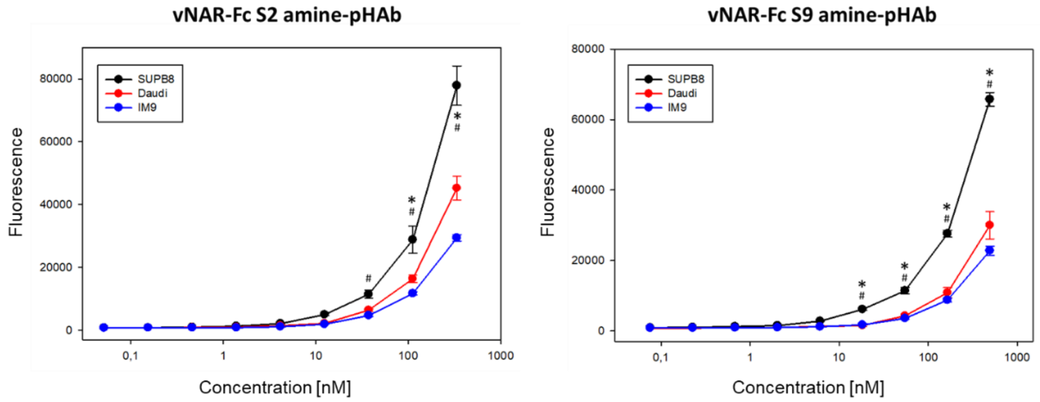

SUPPLEMENTARY FIGURE S11 | Internalization assay of vNAR-Fc fusion proteins. Lymphoma B cells were treated overnight with a concentration series of amine-pHAb conjugated vNAR-Fc antibodies. Endocytosis of pHAb-conjugated antibodies can be quantified by means of increasing fluorescence at acidic pH. vNAR-Fc antibodies were conjugated with a 20 molar excess of pHAb dye and purified using a desalting column. Internalization rates were monitored at a plate reader at Ex/Em of 532 nm/560 nM. Mean ± SEM of triplicates are plotted. Results were analyzed by two-way ANOVA (Bonferroni t-test) and significant differences ($p \leq 0.05$) between on-target SUP-B8 and control Daudi and IM-9 B cells are depicted by * and #, respectively.

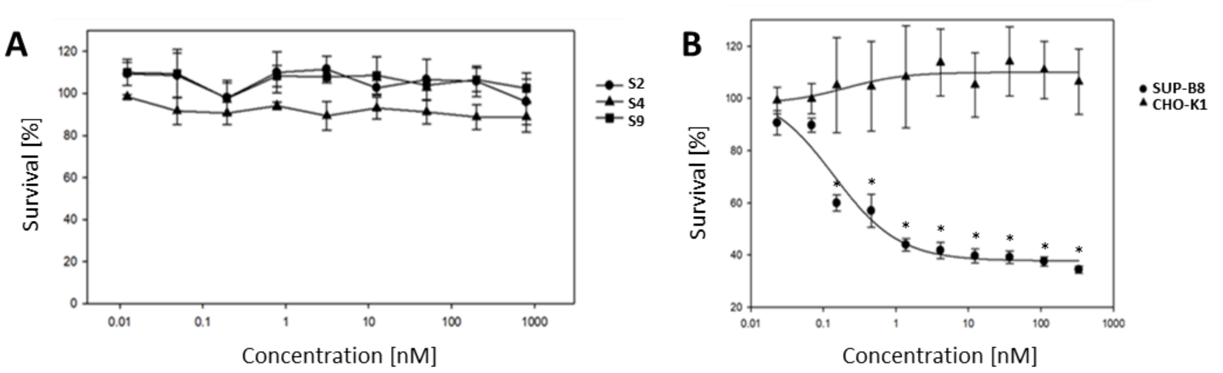

SUPPLEMENTARY FIGURE S12 | Cytotoxicity assays of different vNAR-Fc variants using the MTS Cell Proliferation Assay. B-cell line SUP-B8 was treated with three bivalent vNAR-Fc variants S2, S4 and S9 at varying concentrations (12 pM – 800 nM) **(A)**. Specificity of the vNAR-derived antibody-drug conjugates was assessed upon treatment of on-target SUP-B8 B-cells as well as unrelated CHO-K1 cells with varying concentrations of the MMAE-conjugated vNAR(S9)-Fc antibody **(B)**. Cell proliferation assays were performed in triplicates and the relative survival after 72 h treatment was plotted against the antibody concentrations. Results are shown as mean ± SEM and are representative of at least three independent experiments. Data were analyzed by two-way ANOVA (Bonferroni t-test), and significant differences ($p \leq 0.05$) between control and on-target cell line are depicted by *.

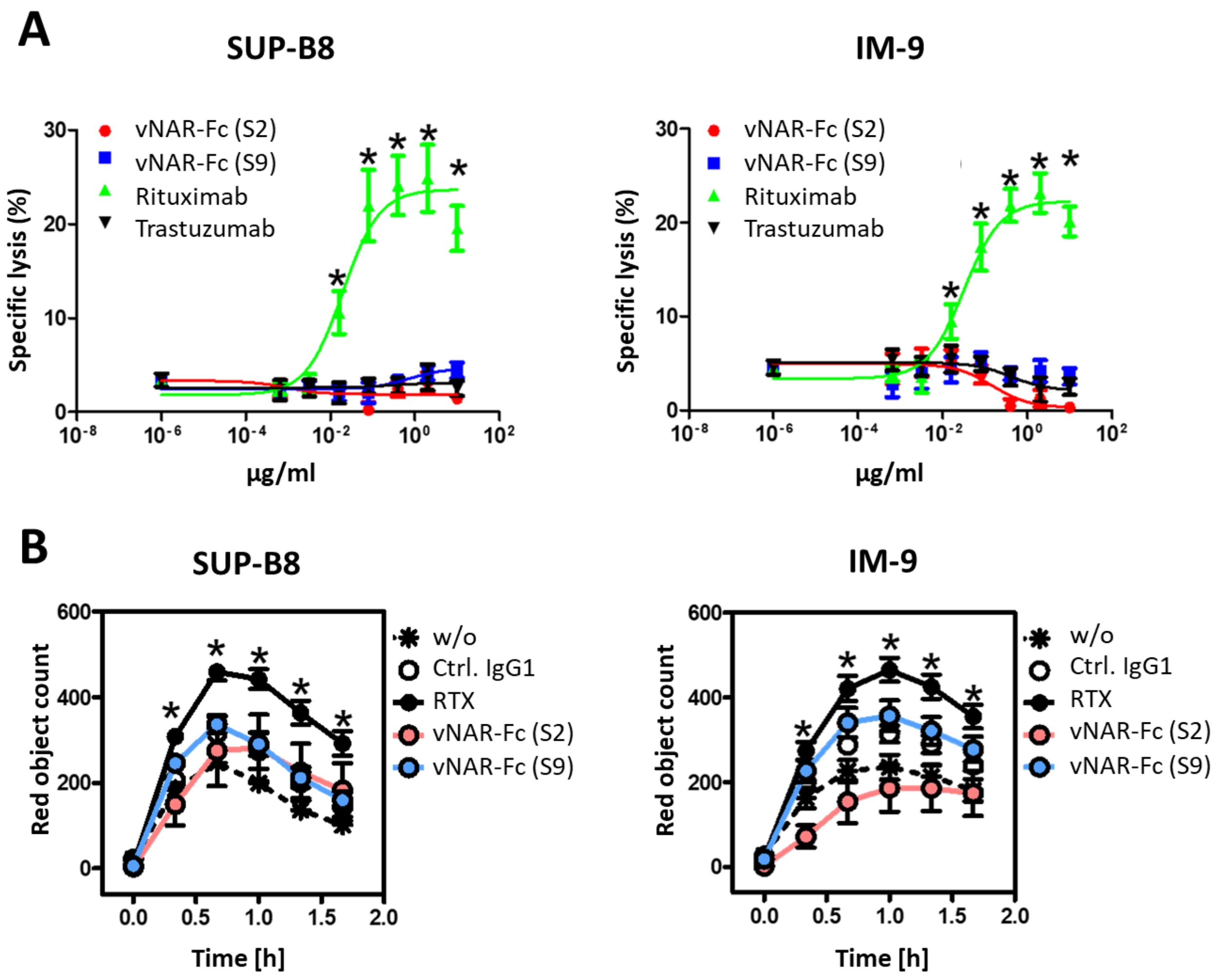

SUPPLEMENTARY FIGURE S13 | Fc-mediated effector functions. Mononuclear cells isolated from human blood served as effector cells in ADCC assays against lymphoma B cells. Effector cells were treated with increasing concentrations of vNAR-Fc fusion proteins and co-cultivated with SUP-B8 (on-target) or IM-9 (off-target) cells for 4 h. Rituximab and trastuzumab were used as positive and negative controls, respectively **(A)**. Human macrophages served as effector cells against malignant B cells in ADCP assays. In ADCP assays, human macrophages were used as effector cells against malignant B-cells. After treatment with vNAR-Fc fusion protein, rituximab or a control IgG1 antibody, macrophages were co-cultivated for 2 h with lymphoma B cells **(B)**. Results are shown as mean ± SEM obtained from 3 independent experiments (3 effector independent donors). Data were analyzed by two-way ANOVA, and significant differences ($p \leq 0.05$) between control and specific antibodies are depicted by *.

2 Supplementary Tables

SUPPLEMENTARY TABLE S1 | Oligonucleotide primers used in this study

Name	Sequence (5' – 3')
Amplification of BCR V_H and V_L	
VH1-5'clon	CAGGTGCAGCTGGTGCAGTCTGG
VH2-5'clon	CAGGTCACCTTGAAGGAGTCTGG
VH3-5'clon	GAGGTGCAGCTGGTGGAGTCTGG
VH4-5'clon	CAGGTGCAGCTGCAGGAGTCGGG
VH5-5'clon	GAGGTGCAGCTGGTGCAGTCTGG
VH6-5'clon	CAGGTACAGCTGCAGCAGTCAGG
Cμ-3'	CTCTCAGGACTGATGGGAAGCC
Cμ-clon	GGAGACGAGGGGGAAAAG
IgG-3'	GCCTGAGTTCCACGACACC
IgG-clon	CAGGGGGAAGACCGATGG
Vκ1-5'clon	GACATCCAGATGACCCAGTCTCC
Vκ2/3-5'clon	GATATTGTGATGACCCAGACTCCA
IgκC-3'	CCCCTGTTGAAGCTCTTTGT
IgκC-clon	AGATGGCGGGAAGATGAAG

VL1_(51)_clon	CAGTCTGTGTTGACGCAGCCGCCCTC
VL1_(36-47)_clon	TCTGTGCTGACTCAGCCACCCTC
VL1_(40)_clon	CAGTCTGTCGTGACGCAGCCGCCCTC
VL2-clon	TCCGTGTCCGGGTCTCCTGGACAGTC
VL3-clon	ACTCAGCCACCCTCGGTGTCAGTG
VL4-clon	TCCTCTGCCTCTGCTTCCCTGGGA
VL5-clon	CAGCCTGTGCTGACTCAGCC
IGLC-3’	GTGTGGCCTTGTTGGCTTG
IGLC2-7_clon	CGAGGGGGCAGCCTTGGG
IGLC1_clon	AGTGACCGTGGGGTTGGCCTTGGG
Cloning for Production of B-Cell Receptors: Variable heavy chain	
Daudi_HC_BamHI_up	GCTGATGTTCTGGATCCCTGCTAGCTTAAGCGAGGTGCAGCTGGTGGAGTCTGG
Daudi_HC_ApaI_lo	GGGAAGACCGATGGGCCCTTGGTGGAGGCTGAGGAGACGGTGACCAG
IM9_HC_ BamHI_up	GCTGATGTTCTGGATCCCTGCTAGCTTAAGCGAGGTGCAGCTGGTGGAGTCTGGG GGAGGC
IM9_HC_ ApaI_lo	GGGAAGACCGATGGGCCCTTGGTGGAGGCTGAAGAGACGATGACCAT
SUPB8_HC_ BamHI_up	GCTGATGTTCTGGATCCCTGCTAGCTTAAGCCAGATTCAGCTGGTGCAGTCTGGA GGTGAG

SUPB8_HC_ ApaI_lo	GGGAAGACCGATGGGCCCTTGGTGGAGGCTGAGGAGACGGTGACCA
Cloning for Production of B-Cell Receptors: Variable light chain	
Daudi_LC_BamHI_up	GCTGATGTTCTGGATCCCTGCTAGCTTAAGCGAGCTCCAGATGACCCAGTCTCCATCCTCC
Daudi_LC_SOE_LO	GAAGACAGATGGTGCAGCCACAGTTCGTTTGTTGTCCACCTT
Daudi_LC_SOE_UP	AAGGTGGACAACAAACGAACTGTGGCTGCACCATCTGTCTTC
IM9_LC_BamHI_up	GCTGATGTTCTGGATCCCTGCTAGCTTAAGCGAGCTCCAGATGACCCAGTCTCCTTCCACCCTG
IM9_LC_SOE_lo	GAAGACAGATGGTGCAGCCACAGTTCGTTTGATTTCCACCTTGGT
IM9_LC_SOE_up	ACCAAGGTGGAAATCAAACGAACTGTGGCTGCACCATCTGTCTTC
SUPB8_Lambda_NotI_lo	CCTACAGAATGTTCGTAATAGGCGGCCGC AGATCCCCCGACCTC
SUPB8_LC_BamHI_up	GCTGATGTTCTGGATCCCTGCTAGCTTAAGCCAGTCTGTGTTGACGCAGCCG
SUPB8_LC_SOE_lo	TGGCGGGAACAGAGTGACCGAGGGGGCAGCCTTGGG
SUPB8_LC_SOE_up	CCCAAGGCTGCCCCCTCGGTCACTCTGTTCCCGCCA
SUPB8_Lambda_NotI_lo	GAGGTCGGGGGATCTGCGGCCGCCTATTACGAACATTCTGTAGG
Sequencing	
pCT_seq_up	TACCCATACGACGTTCCAGACTAC
pCT_seq_lo	CAGTGGGAACAAAGTCGATTTTGTTAC

pExp_seq_up	GAGAACCCACTGCTTACTGGC
pExp_seq_lo	CACGCCGTCCACATACCAGTTGAAC
Expression	
pExp_NheI_up	GCGCGCGCTAGCCGCTGAGAACCTGTACTTCCAGAGCGCCGTGACGTTGGACGAG
pExp_F1_lo	GGTGTGGGTCTTGTCGCAGCTCTTGGGCTCGCTTCCGCTCTGGAAGTACAGGTTCT CTTTCACAGTCAGAGTGGTGCCGCCTCC
pExp_F2_lo	GGTGTGGGTCTTGTCGCAGCTCTTGGGCTCGCTTCCGCTCTGGAAGTACAGGTTCT CTTTCACAGTCAGAGTGGTGCCGCCCCCTTC
pExp_F4_lo	GGTGTGGGTCTTGTCGCAGCTCTTGGGCTCGCTTCCGCTCTGGAAGTACAGGTTCT CTTTCACAGTCAGAATGGTCCCCCCTCC
pExp_F7_lo	GGTGTGGGTCTTGTCGCAGCTCTTGGGCTCGCTTCCGCTCTGGAAGTACAGGTTCT CTTTCACAGTCACAGTGGTGCCCCCACC
pExp_F8_lo	GGTGTGGGTCTTGTCGCAGCTCTTGGGCTCGCTTCCGCTCTGGAAGTACAGGTTCT CTTTCACAGTCAGAGTGGTCCCGCCGCC
pExp_L1_lo	GGTGTGGGTCTTGTCGCAGCTCTTGGGCTCGCTTCCGCTCTGGAAGTACAGGTTCT CTTTCACAGTCACAGTGGTCCCCCCTCC
pExp_L3_lo	GGTGTGGGTCTTGTCGCAGCTCTTGGGCTCGCTTCCGCTCTGGAAGTACAGGTTCT CTTTCACAGTCACAGTGGTGCCGCCACC
pExp_ApaI_lo	GCGCGCGGGCCCGCCCAGCAGTTCAGGGGCAGGGCAGGGAGGACAGGTGTGGGTC TTGTCGCAGCTCTTGGGCTCGCTTCC
